# METTL1 drives tumor progression of bladder cancer via degrading ATF3 mRNA in an m^7^G-modified miR-760-dependent manner

**DOI:** 10.1038/s41420-022-01236-6

**Published:** 2022-11-17

**Authors:** Haiyun Xie, Mingchao Wang, Haifeng Yu, Huan Wang, Lifeng Ding, Ruyue Wang, Wenqin Luo, Zeyi Lu, Qiming Zheng, Liangliang Ren, Zhenwei Zhou, Wenjing Su, Liqun Xia, Gonghui Li

**Affiliations:** 1 Department of Urology, Sir Run Run Shaw Hospital, School of Medicine, Zhejiang University, 310016 Hangzhou, Zhejiang PR China; 2 Life Sciences Institute of Zhejiang University, 866 Yuhangtang Road, 310058 Hangzhou, Zhejiang PR China

**Keywords:** Oncogenes, Methylation

## Abstract

7-methylguanosine (m^7^G) modification is recently found to conservatively exist in RNA internal position besides mRNA caps and mediates the various RNA metabolisms. As the core confirmed transmethylase of m^7^G modification, METTL1 has been reported in certain human cancers. However, the role of internal m^7^G at miRNAs and its core writer METTL1 in bladder cancer (BCa) remains to be elucidated. Here, we demonstrated that METTL1 was indispensable for BCa proliferation and metastasis in vitro and in vivo. By combining miRNA sequencing, m^7^G methylated RNA immunoprecipitation (MeRIP) and RIP, we identified METTL1 promoted the processing of miR-760 in an m^7^G-dependent manner. Transcription sequencing suggested that METTL1 indirectly degrades tumor suppressor ATF3 mRNA mediated by miR-760. Together, we concluded a regulatory axis composed of METTL1/m^7^G/miR-760/ATF3 in regulating BCa progression and provided potential therapeutic targets for BCa.

## Introduction

Bladder cancer (BCa) ranks 10th of most commonly diagnosed cancer and threatens the health of human beings [1] with approximately 573,000 new cases and 213,000 deaths estimated by GLOBOCAN in 2020. It is urgent and also challenging to explore the mechanism of BCa and figure out the potential therapeutic targets.

With the birth of epitranscriptome, kinds of RNA modification and their important roles in cancer are depicted [2]. Modifications exist in every kind of RNA, including messenger RNAs (mRNAs) [3], transfer RNAs (tRNAs), ribosomal RNAs (rRNAs) [4], long noncoding RNAs (lncRNAs) [5], and microRNAs (miRNAs) [6], potentially affecting RNA metabolism and function. For example, N6-methyladenosine (m^6^A) in mRNA is associated with RNA splicing [7], RNA stability [8], and translation efficiency [9]. N5-methylcytosine (m^5^C) in tRNA is related to RNA stability [10] and is related to RNA assembly in rRNA [11]. 5’ phospho-dimethyl modification in miRNA regulates miRNA maturation [6] and m^6^A in miRNA is a key post-transcriptional modification promoting primary miRNA processing [12, 13]. The dysregulation of RNA metabolism induced by RNA modification imbalance are essential to cancers [14, 15].

N7-methyguanosine (m^7^G), first identified at 5’cap structure of mRNA in 1974 [16], is also found in the internal position of RNA recently, including tRNA [17], rRNA [18], internal mRNA [19, 20] and miRNA [21]. METTL1 (methyltransferase-like 1) and WDR4 (WD repeat domain 4), homologous to Trm8 and Trm82 in yeast [22], are presently recognized as internal m^7^G modification “writer” complex. m^7^G at tRNA catalyzed by METTL1/WDR4 was found to stabilize tRNA and increase tRNAs expression, which resulted in enhanced oncogenic mRNA translation and tumor progression in a series of cancers, such as intrahepatic cholangiocarcinoma, glioblastoma multiforme, hepatocellular carcinoma, lung cancer and bladder cancer [23–27]. METTL1/WDR4 also catalyzed m^7^G modification at let-7 miRNA to promoted the tumor suppressor miRNA processing from primary to precursor miRNA in lung cancer [21]. In addition, METTL1/WDR4 was detected to catalyze abundant m^7^G-modified sites at internal mRNA and promote translation efficiency of modified RNA [19].

However, whether METTL1 and RNA m^7^G modification are involved in tumorigenesis of BCa remains to be explored. Here, we investigated that METTL1 was overexpressed and play an oncogenic role in BCa. The proliferation and metastasis of BCa were inhibited upon depletion of METTL1 in vitro and vivo. By miRNA sequencing, RIP-RT-qPCR and MeRIP-RT-qPCR, we found that METTL1 could directly bind and methylate pri-miR-760 to regulate the processing of miR-760. And increased miR-760 degraded tumor suppressor gene ATF3 mRNA by confirmation of dual-luciferase reporter assay. Consequently, we speculated an important regulation axis in BCa that METTL1 promoted the processing of miR-760 in an m^7^G modification-dependent way and contributed to degradation of ATF3 mRNA, accelerating the tumor growth and metastasis eventually. We expected the underlying mechanism of METTL1 and m^7^G modification in miRNA could supply a potential therapeutic target for BCa.

## Results

### METTL1 is overexpressed in BCa tissues and cell lines

**Fig. 1:**
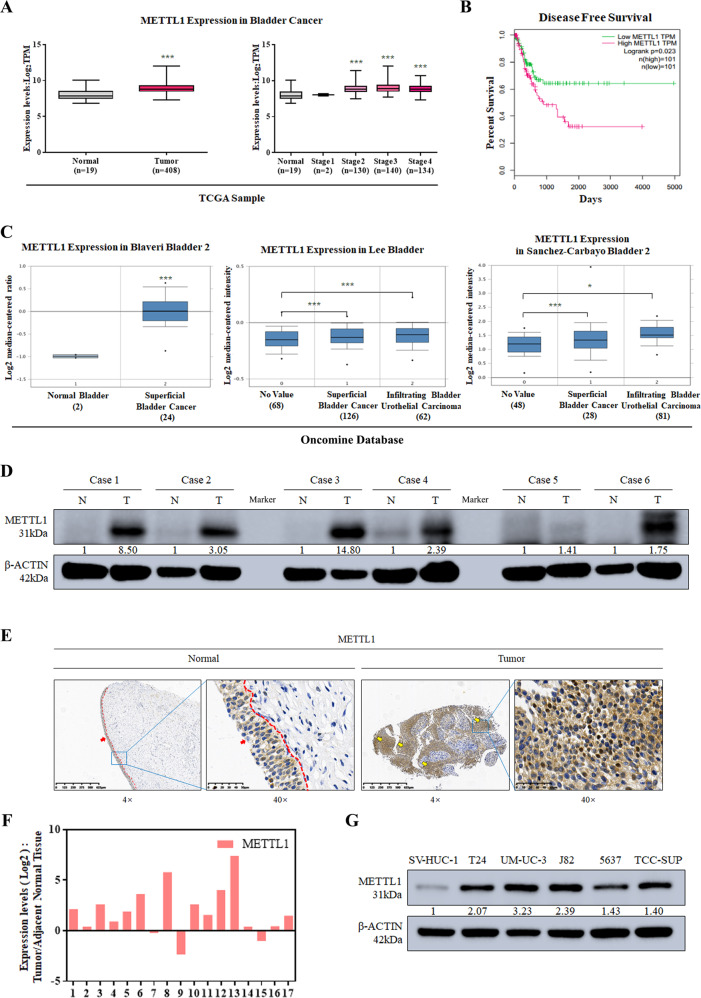
METTL1 is overexpressed in BCa tissues and cell lines. **A** Expression levels of METTL1 in primary tumors vs normal subjects and among stages from TCGA database. Statistical significance was determined by Student’s *t* test: ***P < 0.001. **B** Kaplan–Meier curve revealed a lower disease-free survival rate was observed with the increasing expression level of METTL1 in TCGA database. *P* value (*P* = 0.026) was calculated with log-rank test. **C** Expression levels of METTL1 in Oncomine online database (*P < 0.05, ***P < 0.001). **D** Representative western blot assay showed increased protein expression of METTL1 in patient-derived tumor tissue relative to adjacent normal tissue. β-actin was used for the normalization control. **E** Representative images of IHC staining of METTL1 in patient-derived tissues. Red dotted line was boundary of normal urothelial layer. Red arrows pointed to normal urothelial layer. Yellow arrows pointed to tumor tissues. **F** RT-qPCR assay showed increased mRNA expression level of METTL1 in patient-derived tumor tissue relative to adjacent normal tissue. **G** Western blot assay showed the upregulated protein expression of METTL1 in BCa cancer cell lines (T24, UM-UC-3, J82, 5637, TCC-SUP) compared to the human normal urothelial cell line (SV-HUC-1). β-actin was used for the normalization control. A representative experiment of three independent biological replicates is shown.

METTL1, as an important methyltransferase [28], was reported to be overexpressed in many cancers. To investigate the expression pattern of METTL1 in BCa, TCGA and Oncomine databases were utilized. In the TCGA database, the expression of METTL1 in the primary tumor (*n* = 408) was significantly elevated compared to normal subjects (*n* = 19) (*P* < 0.001) (Fig. 1A). With the expression level increasing, the poorer prognosis was observed (Fig. 1B), and the higher expression of METTL1 was observed in bladder cancer with metastasis group comparing to bladder cancer without metastasis group (*P* value: 2.022e-3) (Supplementary Fig. S1A). Overexpression of METTL1 mRNA was also confirmed in the Blaveri cohort (*P* < 0.001). Furthermore, Lee and Sanchez–Carbayo cohorts showed that the level of METTL1 mRNA was positively correlated with grade malignancy (*P* < 0.05) (Fig. 1C). In addition, we observed upregulated METTL1 at protein expression level in BCa tissues from Human Protein Atlas (Supplementary Fig. S1B). Consistently, in patient-derived BCa tissues from our clinical center (*n* = 17), western blot assay and IHC staining revealed increased expression of METTL1 at the protein level compared with tissues adjacent to cancer, which was also confirmed in mRNA level by RT-qPCR assay (Fig. 1D–F). At the cell level, western blot assay also confirmed that the METTL1 protein expression in BCa cell lines (T24, UM-UC3, J82, 5637, TCC-SUP) was higher than the normal human urothelial cell line SV-HUC-1 (Fig. 1G). These results suggested that METTL1 was elevated in BCa, and its expression level correlated with tumor progression and poor prognosis.

### Depleted METTL1 significantly suppresses the proliferation and migration of BCa in vitro

**Fig. 2:**
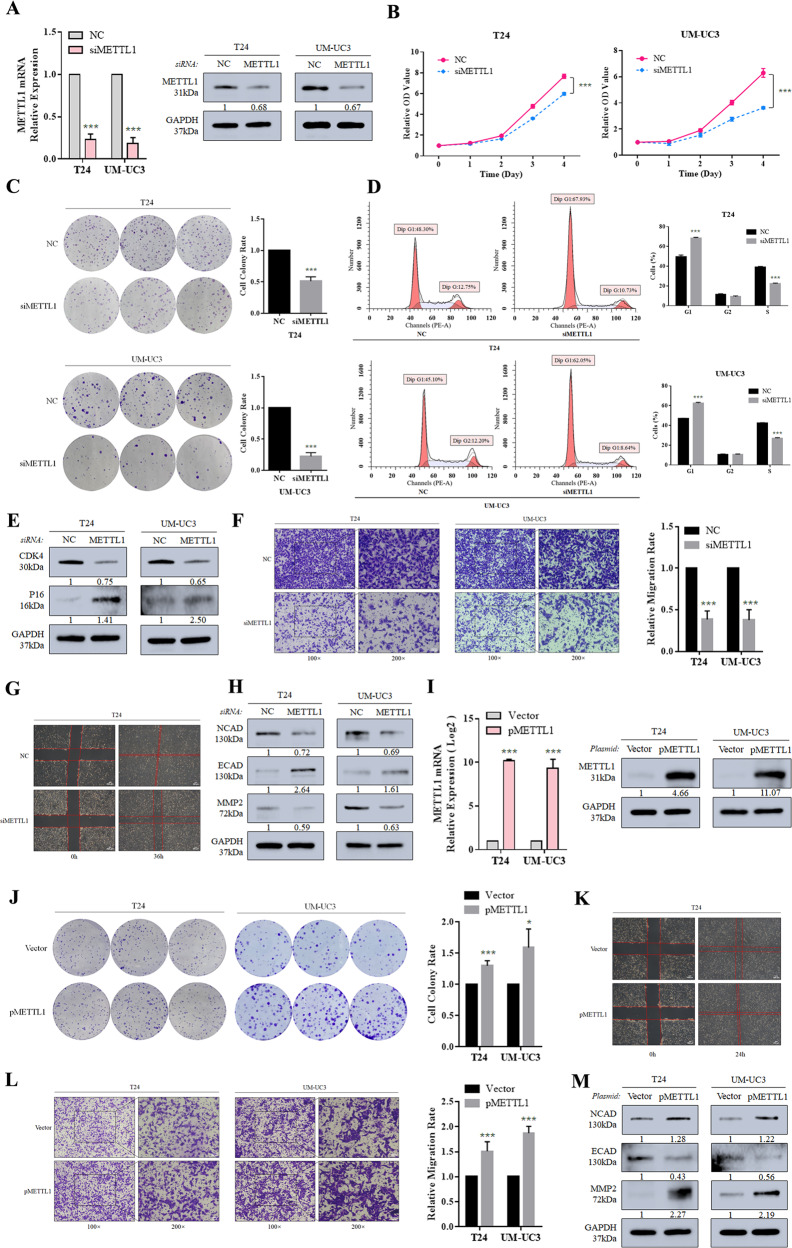
Depleted METTL1 significantly suppresses proliferation and migration of BCa in vitro. **A** RT-qPCR assay and western blot assay showed METTL1 was knocked down by siMETTL1 pool at mRNA and protein expression levels. GAPDH was used for the normalization control. The average of three independent biological replicates ±SDs is shown (***P < 0.001) in RT-qPCR assay, and a representative experiment of three independent biological replicates is shown in western blot assay. **B** Proliferation assay revealed the inhibited proliferation rate of T24 and UM-UC3 upon METTL1 depletion. The average of six biological replicates ±SDs is shown (***P < 0.001). **C** Colony-formation assay showed decreased colony formed upon METTL1 depletion. The average of three independent biological replicates ±SDs is shown (***P < 0.001). **D** Cell cycle analysis indicated increased cells arrested in G1 phase upon METTL1 depletion. The average of three independent biological replicates ±SDs is shown (***P < 0.001). **E** Representative western blots showed cell cycle-associated proteins altered upon METTL1 depletion. A representative experiment of three independent biological replicates is shown. **F** Transwell assay suggested impaired migration ability of METTL1-depleted cells. The average of three independent biological replicates ±SDs is shown (***P < 0.001). **G** Wound-healing assay showed impaired migration ability of METTL1-depleted T24 cells. A representative experiment of three independent biological replicates is shown. **H** Representative western blots showed the alterations of EMT-associated proteins in METTL1-depleted cells. A representative experiment of three independent biological replicates is shown. **I** RT-qPCR assay and western blot assay showed METTL1 was overexpressed by pMETTL1 at mRNA and protein expression levels. The average of three independent biological replicates ±SDs is shown (***P < 0.001) in RT-qPCR assay. A representative experiment of three independent biological replicates is shown in western blot assay. **J** Colony-formation assay showed increased colony formed by METTL1 overexpression. The average of three independent biological replicates ±SDs is shown (*P < 0.05, ***P < 0.001). **K** Wound-healing assay suggested enhanced migration ability by METTL1 overexpression in T24 cells. A representative experiment of three independent biological replicates is shown. **L** Transwell assay indicated enhanced migration ability by METTL1 overexpression. The average of three independent biological replicates ±SDs is shown (*P < 0.05, ***P < 0.001). **M** Western blots showed EMT-associated proteins and MMPs altered by METTL1 overexpression. A representative experiment of three independent biological replicates is shown.

To explore the potential function of METTL1, we depleted METTL1 by using a homolog-specific siRNA-pool which consists of two different targeting oligos (Fig. 2A). When METTL1 depleted, BCa cell lines T24 and UM-UC3 showed impaired proliferation ability (Fig. 2B). Depletion of METTL1 caused decreased colony-formation ability (Fig. 2C). Further cell cycle analysis revealed that METTL1 depletion induced G1 phase arrest (Fig. 2D), which was further confirmed by downregulated protein expression of CDK4 and upregulated protein expression of P16 (Fig. 2E). In addition, Transwell analysis and wound-healing assay showed significant inhibition of cell migration ability in METTL1 knockdown group (Fig. 2F, G and Supplementary Fig. S2A). We further detected epithelial-mesenchymal transition (EMT)-related proteins and observed that E-cadherin (ECAD) was upregulated while N-cadherin (NCAD) and matrix metalloproteinase-2 (MMP2) were downregulated (Fig. 2H). E-cadherin (ECAD) deficiency was a key factor in BCa metastasis [29]. Upregulated ECAD and downregulated NCAD upon METTL1 depletion indicated that epithelial state was activated. A decrease expression of MMP2, which primarily digested solubilized monomers of collagens I, II, and III [30], was a signal of mesenchymal–epithelial transition (MET) [31, 32]. Thus, we supposed that depletion of METTL1 inhibited EMT progression causing the inhibition of cell migration ability.

To further corroborate the role of METTL1 in BCa progression, we tested cell colony-formation ability and migration ability in METTL1 overexpressed cells, which was achieved by overexpression plasmids (pMETTL1) (Fig. 2I). Ectopic expression of METTL1 significantly increased colony numbers in T24 and UM-UC3 cells (Fig. 2J) and caused more efficient cells migration compared with control group (Fig. 2K, L and Supplementary Fig. S2B). Western blot analysis further confirmed that the EMT progression were consistently activated upon METTL1 overexpression (Fig. 2M), which was opposite to METTL1 depletion. Taken together, these findings indicated that METTL1 played an oncogenic role in BCa.

### METTL1 is demanded for tumor proliferation and metastasis of BCa in vivo

**Fig. 3:**
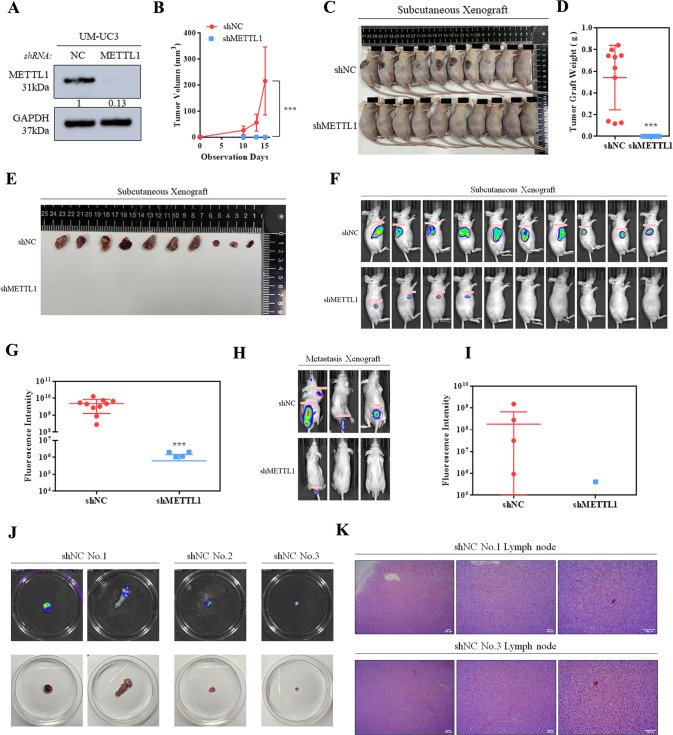
METTL1 is demanded for tumor proliferation and metastasis of BCa in vivo. **A** Western blot assay showed METTL1 was deleted by shMETTL1. GAPDH was used for the normalization control. **B**–**G** Subcutaneous xenograft tumor model (BALB/c nude mice). **B** The tumor growth curve of subcutaneous xenografts plotted by the tumor size (width^2^ × length × 0.52) measured with vernier caliper every 3 days (*n* = 10 each group). **C**–**E** Subcutaneous xenografts in METTL1 depletion group grew too slow to be figured out by the naked eyes at end point of observation. **F**–**J** Fluorescence intensity measured by IVIS system was weaker in shMETTL1 group than shNC group. **H**–**K** Experimental metastases mice model (BALB/c nude mice) by caudal vein injection. **H**, **I** Fluorescence intensity of and the amount of metastasis sites measured by IVIS system decreased in shMETTL1 group. **J** Lymph node metastases and osseous metastases obtained from shNC group. **K** H&E staining of lymph node metastases. The red arrows point to abnormal cells with pathologic mitosis. Statistical significance was determined by Student’s *t* test: ***P < 0.001.

To explore the functional role of METTL1 in vivo, we inactivated METTL1 in UM-UC3 cells (shMETTL1). Cells interfered with three sequences (shMETTL1-1, shMETTL1-2, shMETTL1-3) were detected (Supplementary Fig. S3A–D), and cells interfered with shMETTL1-2, the most effective sequence, was selected to inject into the subcutaneous flank and caudal vein of BALB/c nude mice (4 weeks old) (Fig. 3A). In subcutaneous xenograft mice model, we estimated the proliferation ability by tumor growth rate and final tumor size. We observed xenograft grew slower upon METTL1 deletion (Fig. 3B). The final xenograft size was significantly smaller in the shMETTL1 group compared with shNC group and was too small to be discernible by naked eyes (Fig. 3C–E), which was also proved by fluorescence intensity via imaging system (Fig. 3F, G). Moreover, the metastatic ability of METTL1-depleted UM-UC3 cells was inhibited in experimental metastases mice model by cells caudal vein injection. Both the number of metastases and fluorescence intensity decreased (Fig. 3H–K). In conclusion, METTL1 was indispensable for tumor proliferation and metastasis in vivo.

### METTL1 promotes pri-miR-760 processing in an m^7^G-dependent manner

**Fig. 4:**
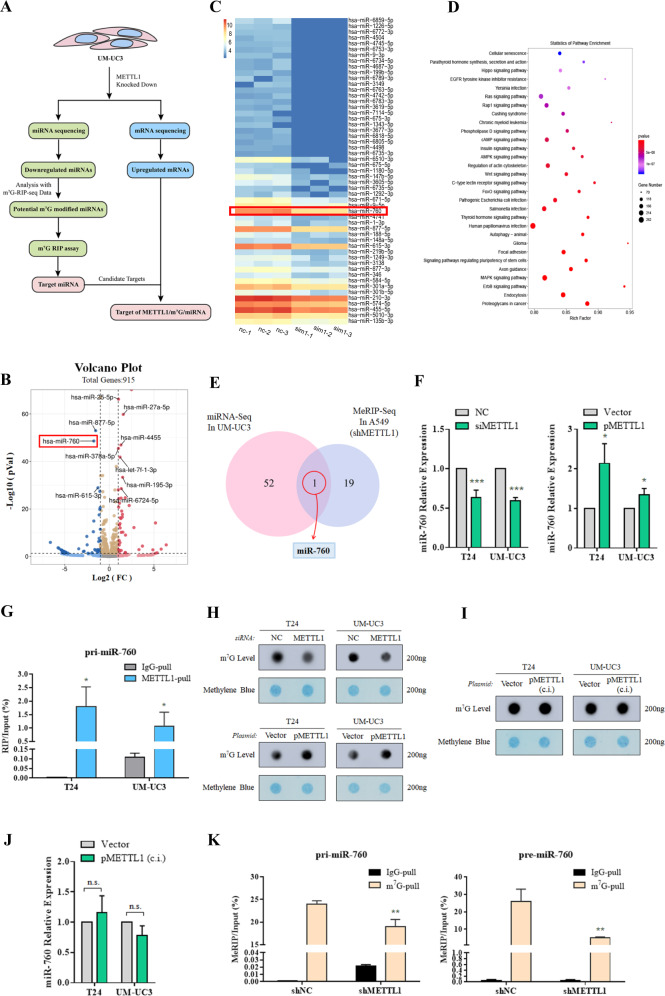
METTL1 promotes miR-760 processing in an m^7^G-dependent manner. **A** Schematic of how to identify downstream targets. **B** Volcano plot showed differentially expressed miRNAs upon METTL1 depletion from miRNA-seq. (|log_2_FC| > 1 and *P* value < 0.05) 53 miRNAs downregulated (blue) and 44 miRNAs upregulated (red). **C** Heatmap showed 53 downregulated miRNA arranged by log_2_FC from smallest. **D** KEGG enrichment analysis of differentially expressed miRNAs upon METTL1 depletion from miRNA-seq. **E** Venn diagram showed the overlap of downregulated miRNAs (log_2_FC < −1 and *P* value < 0.05) in UM-UC3 cells upon the depletion of METTL1 from miRNA-seq and miRNAs downregulated by METTL1 knocked down in A549 cells from MeRIP-seq. **F** RT-qPCR assay showed that downregulated expression of METTL1 led to decreased expression of miR-760 and upregulated expression of METTL1 led to increased expression of miR-760. U6 was used for the normalization control. The average of three independent biological replicates ±SDs is shown (*P < 0.05, ***P < 0.001). **G** Dot blot assay showing downregulation of METTL1 led to decreased m^7^G level and upregulated expression of METTL1 led to increased m^7^G level. **H** Dot blot assay showed m^7^G level in total RNA was unchanged by pMETTL1 with catalytic center inactivation (c.i.). **I** RT-qPCR assay showed the expression of miR-760 was unchanged by pMETTL1 with catalytic center inactivation (c.i.). U6 was used for the normalization control. The average of three independent biological replicates ±SDs is shown. **J** RIP-RT-qPCR assay showed pri-miR-760 was enriched by METTL1 antibody indicating METTL1 bond to pri-miR-760 in T24 and UM-UC3 cells. The average of three independent immunoprecipitation reactions ±SDs is shown (*P < 0.05). **K** MeRIP-RT-qPCR assay showed the decreased pri-miR-760 and pre-miR-760 enriched by m^7^G antibody upon the METTL1 depletion, suggesting the direct methylation of METTL1 in UM-UC3. The average of three independent immunoprecipitation reactions ±SDs is shown (**P < 0.01).

Previous studies have established the significance of miRNA in bladder cancer. METTL1, as an m^7^G writer, was found as a crucial regulator of m^7^G modification on miRNA [33]. To study this connection, we examined METTL1-mediated miRNA expression in BCa (Fig. 4A). To find out the potential miRNAs which can be modified and regulated by METTL1, miRNA sequencing (miRNA-seq) was performed after METTL1 knocked down in UM-UC3 cells. There were 53 miRNAs downregulated and 44 miRNAs upregulated upon METTL1 depletion (Fig. 4B, C). Pathway analysis revealed that the altered miRNAs were involved in some key signaling pathways of cancer such as “MAPK signaling pathway”, “Wnt signaling pathway”, “AMPK signaling pathway”, and “Ras signaling pathway” (Fig. 4D), suggesting METTL1 could regulate tumorigenesis in a miRNA-dependent manner. Referring to the methylated RNA immune-precipitation (MeRIP) sequencing analysis and Borohydride Reduction sequencing analysis (BoRed-seq), which showed 20 miRNAs modified and regulated by METTL1 with a high probability [21], we identified that miR-760 was a potential target of METTL1 in BCa by overlapping the common target miRNAs between Luca’s data and our miRNA-seq data (Fig. 4E). We confirmed that depletion of METTL1 decreased the expression of pre-miR-760 and miR-760 mature forms with no significant changes detected in pri-miR-760, while overexpressed METTL1 increased the expression of miR-760 (Fig. 4F and Supplementary Fig. S4A, B). As METTL1 was confirmed to bind directly to miRNA precursors which includes some primary miRNA hairpins [34], we performed RNA immunoprecipitation experiments in T24 and UM-UC3 cells. Compared with the non-specific IgG, pri-miR-760 and pre-miR-760 were enriched by METTL1-specific antibody, suggesting METTL1 could bind to pri-miR-760 and pre-miR-760 (Fig. 4G and Supplementary Fig. S4C). These results indicated that METTL1 was indispensable for the process of pri-miR-760 to pre-miR-760 processing and might mediate pri-miR-760 processing in an m^7^G modification-dependent manner.

To verify this assumption, we used dot blot assay to detect the m^7^G level upon METTL1 expression alteration. We found that the downregulation of METTL1 led to decreased m^7^G level and overexpression of METTL1 led to increased m^7^G level (Fig. 4H) showing that METTL1 was exactly a m^7^G writer in bladder cancer. Moreover, when overexpressed METTL1 with inactive m^7^G catalytic center (c.i.), m^7^G level and expression of miR-760 stayed unchanged, which suggested METTL1 promoting expression of miR-760 was related with m^7^G catalyzation and the process of pri-miR-760 processing was probably regulated by METTL1 mediated by m^7^G modification (Fig. 4I, J). Using the m^7^G-MeRIP-RT-qPCR, we found that the amount of pri-miR-760 and pre-miR-760 enriched by m^7^G-specific antibody decreased upon METTL1 deletion in UM-UC3 (Fig. 4K). Furthermore, we performed DROSHA IP to clarify whether METTL1 was involved in pri-miR-760 processing. When METTL1 knocked down, we found that the amount of pri-miR-760 enriched by DROSHA-specific antibody reduced comparing to the control group in UM-UC3 cells, indicating METTL1 depletion diminish pri-miR-760 processing activity by DROSHA (Supplementary Fig. S4D). These findings implied that METTL1 directly methylated pri-miR-760 and promoted its processing.

### METTL1 regulates ATF3 expression mediated by the process of miR-760

**Fig. 5:**
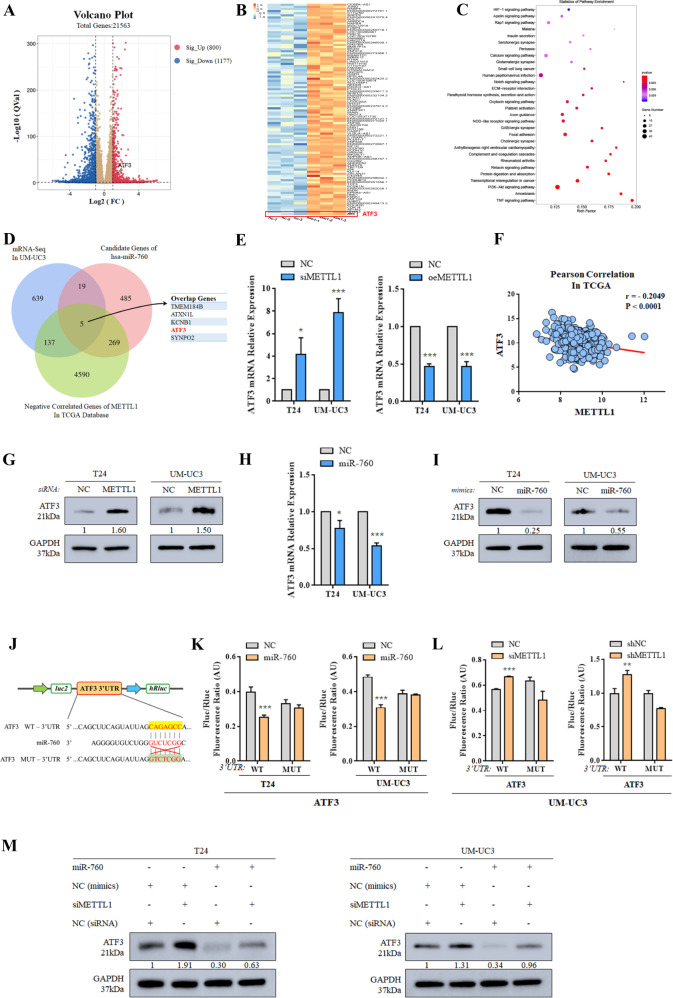
METTL1 regulates ATF3 expression mediated by the process of miR-760. **A** Volcano plot showed differentially expressed mRNAs upon METTL1 depletion from mRNA-seq. (|log_2_FC| > 1, *P* value < 0.05) 800 mRNAs upregulated (red) and 1177 mRNAs downregulated (blue). **B** Heatmap showed a part of upregulated mRNAs from mRNA-seq. **C** KEGG enrichment analysis of differentially expressed mRNAs upon METTL1 depletion from mRNA-seq. **D** Venn diagram showed the overlap among upregulated mRNAs upon METTL1 depletion from mRNA-seq, candidate genes of miR-760 in miRNA online databases and genes negatively correlated with METTL1 in TCGA database. **E** RT-qPCR assay confirmed the expression of ATF3 was negatively correlated with the expression of METTL1. GAPDH was used for the normalization control. The average of three independent biological replicates ±SDs is shown (*P < 0.05, ***P < 0.001). **F** Pearson correlation analysis showed the expression of ATF3 was negatively correlated with the expression of METTL1 in TCGA database. (*r* = −0.2049, *P* value < 0.0001). **G** Western blot assay showed the elevated protein expression of ATF3 upon METTL1 depletion. GAPDH was used for the normalization control. A representative experiment of three independent biological replicates is shown. **H** RT-qPCR assay showing ATF3 was downregulated by overexpression of miR-760. The average of three independent biological replicates ±SDs is shown (*P < 0.05, ***P < 0.001). **I** Western blot assay showing ATF3 was downregulated by overexpression of miR-760 at protein expression level. GAPDH was used for the normalization control. A representative experiment of three independent biological replicates is shown. **J** Schematic showing the target site of miR-760 in 3’UTR of ATF3 mRNA was muted as presented. **K** Dual-luciferase reporter assay indicated miR-760 significantly suppressed the luciferase activity of vectors carried 3’UTR of ATF3. The average of three independent biological replicates ±SDs is shown (***P < 0.001). **L** Dual-luciferase reporter assay indicated depleted METTL1 enhanced luciferase activity of vectors carried 3’UTR of ATF3, which was targeted by miR-760 in UM-UC3. The average of three independent biological replicates ±SDs is shown (**P < 0.01, ***P < 0.001). **M** Western blot assay showing the rescue of ATF3 upregulation upon transfection with miR-760 in METTL1-depleted cells. A representative experiment of three independent biological replicates is shown.

It is well known that miRNAs bind to 3’UTR of their target mRNAs and regulate mRNA stability and translational silencing. Thus, we supposed that METTL1 could regulate gene expression through modulating m^7^G of miR-760. To further figure out downstream targets, we used transcriptome sequencing (mRNA-seq) in METTL1 knocked down UM-UC3. When METTL1 was silenced, 800 genes were upregulated and 1177 genes were downregulated (Fig. 5A, B). These altered genes concerned “PI3K-Akt signaling pathway”, “transcriptional misregulation in cancer”, and “small cell lung cancer”, suggesting the significant regulation role of METTL1 in BCa (Fig. 5C). To identify the direct target of miR-760, online prediction websites including Targetscan, miRDB and miRWalk were fully used. By overlapping mRNA-seq result and TCGA database, we found five genes which were potential targets of the METTL1/m^7^G/miR-760 axis (Fig. 5D). By confirming the expression changes of these five genes at RNA expression level, the mRNA level of ATF3 was significantly elevated when METTL1 was depleted and finally chosen as the potential best-matched target of METTL1/m^7^G/miR-760 (Fig. 5E and Supplementary Fig. S5A–C). In TCGA database, the expression of ATF3 was negatively correlated with METTL1 (*r* = −0.2049, *P* < 0.0001) (Fig. 5F). We also found elevated ATF3 at protein level in METTL1-depleted BCa cell lines (Supplementary Fig. 5G). In addition, ATF3 mRNA expression and protein expression were downregulated by transfection of miR-760 mimics, suggesting that ATF3 mRNA was a potential target of miR-760 and could be degraded by miR-760 (Fig. 5H, I). Therefore, we predicted the direct binding site of miR-760 in 3’UTR of ATF3 mRNA by Targetscan and constructed the wild type sequence (WT) or muted sequence (MUT) into dual-luciferase reporter vector (Fig. 5J). In the dual-luciferase reporter assay, overexpression of miR-760 decreased the luciferase activity of wild type group, proving that the site in 3’UTR of ATF3 mRNA was one of miR-760 targets (Fig. 5K). In addition, the depletion of METTL1 increased the luciferase activity of wild type group, confirming that METTL1 could degrade ATF3 mRNA in a miR-760-dependent way (Fig. 5L). Rescue experiments in western blot assay further demonstrated the upregulated expression of ATF3 upon METTL1 depletion decreased by overexpression of miR-760 (Fig. 5M and Supplementary Fig. S6A), showing METTL1 and miR-760 shared same target in ATF3 mRNA. In conclusion, we assumed that METTL1 accelerated the degradation of ATF3 mRNA mediated by modifying pri-miR-760 and promoting the processing of miR-760.

### METTL1/m^7^G/miR-760/ATF3 regulation axis promotes proliferation and migration of BCa

**Fig. 6:**
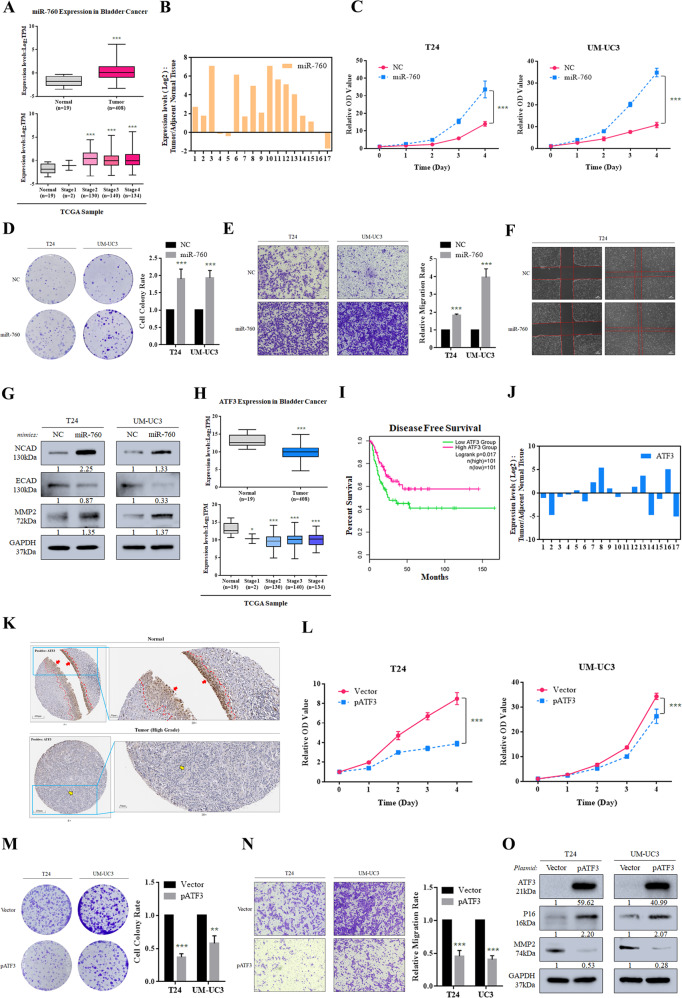
METTL1/m^7^G/miR-760/ATF3 regulation axis promotes proliferation and migration of BCa. **A** Expression levels of miR-760 in primary tumors vs normal subjects and among stages from TCGA database (***P < 0.001). **B** RT-qPCR assay showing expression level of miR-760 in patient-derived tumor tissue relative to adjacent normal tissue. GAPDH was used for the normalization control. **C** Increased proliferation rate of T24 and UM-UC3 by overexpression of miR-760. The average of six biological replicates ±SDs is shown (***P < 0.001) **D** Representative colony-formation assay showing increased colony formed by overexpression of miR-760. The average of three independent biological replicates ±SDs is shown (***P < 0.001). **E** Tanswell assay indicating the enhanced migration ability of cells overexpressed miR-760. The average of three independent biological replicates ±SDs is shown (***P < 0.001). **F** Wound-healing assay indicating the enhanced migration ability of cells overexpressed miR-760 in T24 cells. A representative experiment of three independent biological replicates is shown. **G** Western blots showing the alteration of EMT-associated proteins by miR-760 overexpression. GAPDH was used for the normalization control. A representative experiment of three independent biological replicates is shown. **H** Expression levels of ATF3 in primary tumors vs normal subjects and among stages from TCGA database (*P < 0.05, ***P < 0.001). **I** Kaplan–Meier curve revealed a higher disease-free survival rate was observed with the increasing expression level of ATF3 in TCGA database. *P* value (*P* = 0.017) was calculated with log-rank test. **J** RT-qPCR assay showing expression level of ATF3 in patient-derived tumor tissue relative to adjacent normal tissue. GAPDH was used for the normalization control. **K** IHC staining of ATF3 in patient-derived normal tissue and tumor tissue from the Human Protein Atlas. **L** Decreased proliferation rate of T24 and UM-UC3 by overexpression of ATF3. The average of six biological replicates ±SDs is shown (***P < 0.001) **M** Representative colony-formation assay indicating decreased colony formed by overexpression of ATF3. The average of six biological replicates ±SDs is shown (***P < 0.001). **N** Tanswell assay indicating impaired migration ability in ATF3 overexpressed cells. **O** Western blots showing cell cycle-associated protein and EMT-associated protein altered by overexpression of ATF3. GAPDH was used for the normalization control. A representative experiment of three independent biological replicates is shown.

As proved above that miR-760 was a target of METTL1, we assumed that miR-760 played an oncogenic role in BCa. In the TCGA database, elevated expression of miR-760 was observed in primary tumor tissues (*n* = 408) compared with normal tissues (*n* = 19) (*P* < 0.0001) (Fig. 6A), which was consistent with the expression in patient-derived tissues (*n* = 17) (Fig. 6B). To figure out the functional role of miR-760 in BCa, we tested proliferation and migration ability in miR-760 overexpressed cells. Accelerated proliferation rate and increased colony formation were observed after cells were transfected with miR-760 mimics (Fig. 6C, D). And transfection of miR-760 mimics contributed to more cells migrated and progression of EMT process (Fig. 6E–G and Supplementary Fig. S2C). In addition, inhibited proliferation and migration of METTL1 depletion were rescued by miR-760 (Supplementary Fig. S6B, C). Taken together, it was inferred that enhanced proliferation and migration ability induced by METTL1 was partially attributed to miR-760.

ATF3, an important transcription factor, was found to suppress metastasis of BCa by regulating the actin cytoskeleton [35]. Compared to normal tissues (*n* = 19), ATF3 at a higher expression level in primary tumor tissues (*n* = 408) was along with a higher disease-free survival rate in the TCGA database (Fig. 6H, I). And the downregulated expression of ATF3 was detected in patient-derived tissues by RT-qPCR assay and western blot assay (Fig. 6J and Supplementary Fig. S7A), which was also confirmed at protein expression level by IHC staining in BCa tissues from the Human Protein Atlas (Fig. 6K). As ATF3 chosen for a target of METTL1/ m^7^G/ miR-760 regulation axis, we made a simple functional confirmation in T24 and UM-UC3 cells. The overexpression of ATF3 significantly repressed the proliferation and migration of BCa by influencing cell cycle-associated protein and EMT-associated protein (Fig. 6L–O). Consequently, the tumor suppressor role of ATF3 made a further statement that ATF3 was a target gene regulated by METTL1-modified miR-760.

**Fig. 7:**
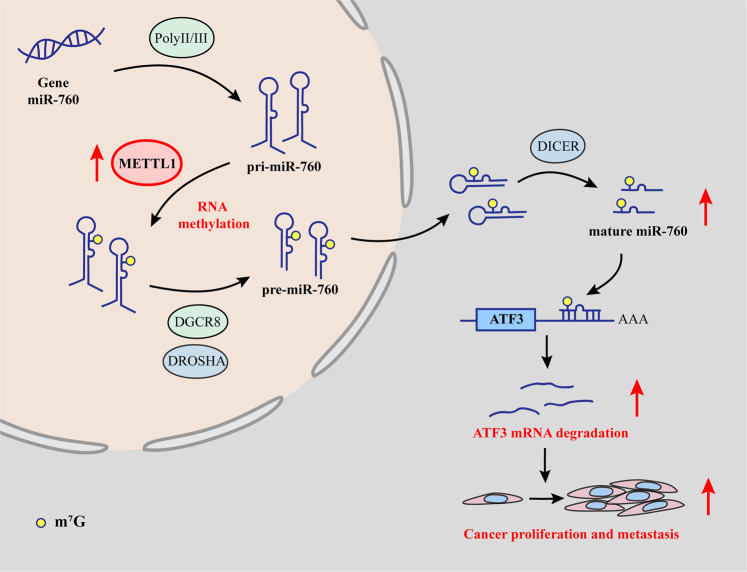
Schematic of all findings in this study. m^7^G modification of pri-miR-760 catalyzed by METTL1 promoted the processing of miR-760, which degraded ATF3 mRNA, driving cancer proliferation and cancer metastasis of BCa.

In conclusion, the oncogenic role of miR-760 and tumor suppressor role of ATF3 help to explain the potential regulatory mechanism of METTL1 in BCa. We summarized a regulatory axis that METTL1 promoted the processing of miR-760 by m^7^G modification to accelerate the degradation of ATF3, which made a progression of proliferation and migration in BCa (Fig. 7).

## Discussion

Limited by detecting techniques, internal RNA m^7^G modification and its functional role in cancers have been discovered in recent years. By far the “writer” complex is found composed of METTL1 and WDR4 [22]. And in many kinds of cancer, imbalance of internal RNA m^7^G modification induced by dysregulation of METTL1 or WDR4 played an important role in tumorigenesis [36]. Upregulated METTL1 and WDR4 promoted the translation of oncogenic transcripts depending on m^7^G-modified tRNA to promote intrahepatic cholangiocarcinoma progression [23]. And m^7^G tRNA modifications and m^7^G codon usage mediated by promote METTL1/WDR4 oncogenic mRNA translation to facilitate lung cancer progression [37]. However, the exact mechanism of internal RNA m^7^G modification mediated by METTL1/WDR4 in BCa still needs exploring.

In this study, we demonstrated that METTL1 was overexpressed in BCa and the high expression level was correlated with poor prognosis. With METTL1 depletion in vitro, BCa cell lines were stuck in the G1 phase of cell cycle and failed to proliferate. Simultaneously, cells failed to migration caused by EMT progression inhibition. In vivo, tumor growth and metastasis were significantly suppressed upon METTL1 deletion. Referring to the identified mechanism that METTL1 could modify miRNA and promote it processing [21] and the significant roles of miRNA found in BCa [38, 39], we performed miRNA sequencing in METLL1-depleted UM-UC3 to figure out the potential m^7^G modified miRNAs. By synthetically analyzing the sequencing results combined with MeRIP-RT-qPCR and RIP-RT-qPCR confirmation, METTL1 was identified to modified m^7^G in G-rich miRNA miR-760 and regulated the processing of it.

miRNA, generally consisting of 20–22 nucleotides, is one of the important noncoding RNA extensively involved in BCa [40]. It was reported that miRNAs targeted 3’UTR of mRNAs to degrade them and/or decreased translation efficiency of them [41]. Considering the mRNA regulating effect of miRNA and miRNA regulating effect of METTL1, we screened genes negatively correlated with METTL1 and confirmed tumor suppressor ATF3 was the downstream target of METTL1-modified miR-760, which was confirmed by dual-luciferase reporter assay. Thus, we described a regulatory axis that METTL1 promoted the processing of miR-760 in an m^7^G-dependent manner to degrade ATF3 in BCa.

miR-760, identified in 2006 [42], has been reported to play a regulatory role in many kinds of cancers [43]. In breast cancer, miR-760 was downregulated in stem cell of BT-549 and regulated tumor metastasis [44]. In ovarian cancer, upregulated miR-760 enhanced cell proliferation and migration [45]. In our confirmation, miR-760 upregulated in BCa tissues promoted cell proliferation and migration. As a result, it could be inferred that the overexpressed METTL1 made miR-760 increased causing tumor promoting.

ATF3 is a crucial transcription factor, regulating some well-known genes such as p53, p21 and influencing some important pathway such as NF-κb, Smad, ERK, JNK, and p38 pathways. The role of ATF3 depends on different kind of cancers, acting tumor suppressor or tumor promotor [46]. In BCa, ATF3 has been found to regulate gelsolin-mediated remodeling of actin cytoskeleton to suppress tumor metastasis [35] amd can be utilized as a potential marker of the response to histone deacetylase inhibitor mediated therapy in bladder cancer [47]. And in this study, we confirmed the tumor-suppressive role of ATF3 in BCa, showing that overexpression of ATF3 repressed the proliferation and migration of BCa cells.

## Conclusion

In summary, we concluded a regulatory axis composed of METTL1/m^7^G/ miR-760/ATF3. We proved that METTL1 overexpressed in BCa caused miR-760 expression elevated by m^7^G modification and degraded ATF3 mRNA in an m^7^G modified miR-760-dependent manner, promoting proliferation and metastasis of BCa.

## Materials and methods

### Patients and tissue specimens

We used 17 pairs of tumor and adjacent tissue samples obtained from tissue bank of Sir Run Run Shaw Hospital School of Medicine ZheJiang University. Baseline information of 17 patients with BCa was listed in Supplementary Table S1. All samples were collected with informed consent according to the Internal Review and the Ethics Board of Sir Run Run Shaw Hospital (IRB number: 20210301-30).

### Cell lines and cell culture

The human BCa cell lines (T24, UM-UC-3), the normal human urothelial cell line (SV-HUC-1) and HEK293T cells were obtained from the Cell Bank of Type Culture Collection of the Chinese Academy of Sciences and verified by short tandem repeat DNA profiling analysis. SV-HUC-1 was cultured in F12K medium (CR21700, Cienry); T24 was cultured in RPMI1640 medium (CR31880S, Cienry); UM-UC3 was cultured in MEM medium (CR41500S, Cienry); HEK293T was cultured in DMEM medium (CR12800S, Cienry). All mediums were supplemented with 10% FBS and penicillin–streptomycin solution.

### Reagents and transfection

siMETTL1 and miR-760 mimics were synthesized by RiboBio (Guangzhou, China), of which transfection was achieved by lipo2000 (Invitrogen) and jetPRIME (#114-15, Polyplus transfection); pMETTL1 and pATF3 were obtained from Genecopia, of which transfection was achieved by lipo3000 (Invitrogen). pMETTL1 with catalytic center inactivation (c.i.) was constructed according to the sequence from Luca [21]. To avoid off-target effects, two different siRNAs of genes were merged as the siRNA pool to transfect cells in all interference experiments. The sequences are listed: siMETTL1-1: GGGCTGGTGTATACCATAA; siMETTL1-2: GGATGTGCACTCATTTCGA.

### Lentivirus packaging and infection

For lentivirus packaging, HEK293T cells were transfected with lentiviral vector psi-LVRU6GP for METTL1 depletion mixed with the packaging plasmids PAX2 and pMD2.G. The ratio of the three compositions was 2:2:1. At 48 h and 72 h after transfection, the supernatants were harvested and filtrated with 0.45-μm filters. Cells were infected with filtered solution and cultured in puromycin added medium (2 μg/mL) for 7 days to select the infected cells. Three interference sequences are listed and shMETTL1-2 was most effective to be selected to perform animal experiments and MeRIP assay: shMETTL1-1: CCTTCCTAACTTCTTCTACAA; shMETTL1-2: GGGAAGAAAGTTCTACGTAAT; shMETTL1-3: GCTCAATTACCACTTCTGTTT.

### Immunohistochemistry (IHC) staining

Immunohistochemistry (IHC) analysis was performed in patient-derived tissues. The tissues were fixed in paraformaldehyde and embedded in paraffin followed by dewaxing and rehydrating. After blocking with BSA (Sango Biotech, Shanghai, China), the slides were incubated with anti-METTL1 (#14994-1-AP, Proteintech) and anti-ATF3 (#ab254268, Abcam) overnight at 4 °C followed by a secondary antibody incubation for 1 h at room temperature. DAB solution was used for brown color development.

### RNA m^7^G dot blot assay

RNA m^7^G dot blot assay was performed to analyze the altered m^7^G level in total RNA. The specific procedure was described in a previous study [48]. RNA isolated from transfected cells or tissues using RNAiso plus (#9109, Takara) was incubated in mixture of MOPS, Formamide and Formaldehyde to remove the secondary structure. In total, 200 ng of treated RNA samples mixed up with ice-cold 20 × SSC Solution (#S6639-1L, Sigma-Aldrich) were loaded on N+ membrane (#RPN303B, GE health). After UV cross-linked, the membrane was stained by 0.02% methylene blue (#M9140-25G, Sigma-Aldrich). After washing three times in TBST, the membrane was blocked in 5% milk for one hour and incubated with m^7^G antibody (#RN017M, MBL) overnight. The membrane was exposed with imager (Biorad ChemiDoc MP, Biorad) on the second day after HRP-conjugated secondary antibody incubation.

### m^7^G-RNA immunoprecipitation assay

m^7^G-RNA immunoprecipitation assay (MeRIP) was performed according to the protocol from Luca [21]. Briefly, total RNA was extracted from 2 × 10^7^ cells and decapped with CapCLIP Acid Pyrophosphatase (#C-CC15011H, CellScript). The decapped RNA was incubated with 10 μg anti-m^7^G (#RN017M, MBL) or IgG-loaded magnetic beads (#16-663, Sigma-Aldrich). The enriched RNA was purified by RNA Clean & Concentrator-25 (#R1017, Zymo) and evaluated by RT-qPCR.

### RNA immunoprecipitation assay

RNA immunoprecipitation assay was manipulated according to the instruction of Magna RIP Kit (#17-700, Merck Millipore). Approximately, 2 × 10^7^ cells were lysed and RNA were enriched by 10 μg anti-METTL1 antibody (#588192, MRC PPU), anti-DROSHA antibody (#3364, Cell Signaling Technology) or IgG-loaded beads. The enriched RNA was purified and evaluated by RT-qPCR.

### RNA isolation and RT- qPCR

RNA was extracted with RNAiso plus (#9109, Takara). PrimeScript RT reagent Kit (#RR036A, Takara) was used for reverse transcription of total RNA, including pri-miR-760. miRNA 1st Strand cDNA Synthesis Kit (by stem-loop) (#MR101-01, Vazyme) was used for reverse transcription of miRNA, including pre-miR-760 and miR-760. And One Step PrimeScript miRNA cDNA Synthesis Kit (#638315, Takara) was also used for miRNA reverse transcription. Quantitative PCR (qPCR) was performed by UltraSYBR One Step RT-qPCR Kit (#CW2624, CWBIO) in thermocycler (CFX96 Touch Real-Time PCR, Biorad). GAPDH and U6 were chosen as the endogenous reference for calculating the relative expression. All primers are listed in Supplementary Table S2.

### Cell proliferation assay

In all, 2 × 10^4^ treated cells (transfected with siRNAs or plasmids for 48 h) were seeded in 96-well plates. Cell count was evaluated by cell counting kit 8 test (#CK04, Dojindo) for the following 4 days.

### Colony-formation assay

The specific procedure referred to a previous study [48].

### Transwell assay

The specific manipulations were the same as the previous study [48]. Transwell chamber (#3422, Corning) with nearly 3 × 10^4^ transfected cells in 0.3 mL serum-free medium was immersed in 0.8 mL medium with 10% FBS. After 24 h, migrated cells were fixed with methanol, stained with 0.3% crystal violet, and observed by microscope (IX71, Olympus).

### Wound-healing assay

The specific manipulations were the same as the previous study [48]. A cross-shaped break was created by pipette tip on cell layer. Cells were cultured in a serum-free medium to exclude the influence of cellular proliferation and the cellular migration process was monitored by a phase-contrast microscope (IX71, Olympus).

### Cell cycle analysis

Cell cycle analysis of treated cells (transfected with siRNAs for 48 h) was performed as previously described [48]. DNA staining of transfected cells was performed using cell cycle staining Kit (#CCS012, Multi Sciences). The stained cells were analyzed by flow cytometry (the BD LSRII Flow Cytometer System, BD Biosciences, Franklin Lakes, NJ) and the data were analyzed by ModFit LT 3.2 software (Verity Software House, Topsham, ME).

### Western blot assay

Western blot assay was performed as the previous study described [48]. Total proteins were extracted using RIPA buffer (#FD011, Hangzhou Fude Biological Technology) and quantified by BCA protein assay kit (#P0011, Beyotime). In total, 1 μg of proteins were separated by 10% SDS-PAGE and transferred to PVDF membrane (#IPVH00010, Merck Millipore). After blocked with 5% Non-Fat Milk and incubated with specific antibody at 4 °C overnight, followed by HRP-conjugated secondary antibody incubation, the membrane was imaged with imager (Biorad ChemiDoc MP, Biorad). The antibodies used in this study were listed: anti-GAPDH (#5714, Cell Signaling Technology), anti-METTL1 (#ab157097, Abcam), anti-E-cadherin (#20874-1-AP, Proteintech), anti-N-cadherin (#66219-1-AP, Proteintech), anti-VIMENTIN (#10366-1-AP, Proteintech), anti-MMP2 (#10373-2-AP, Proteintech), anti-CDK4 (#11026-1-AP, Proteintech), anti-P16 (#18769, Cell Signaling Technology), anti-ATF3 (#ab254268, Abcam).

### mRNA sequencing and small RNA sequencing

mRNA sequencing was performed as we previously stated [48]. As for small RNA sequencing, total RNA was isolated from UM-UC3 cells using the MagZol (Magen) according to the manufacturer’s protocol. After the quantity and integrity of RNA yield being assessed by K5500 and Agilent 2200 TapeStation (Agilent Technologies, USA) separately, RNAs were ligated with 3’RNA adapter followed by 5’ adapter ligation. Subsequently, the adapter-ligated RNAs were subjected to RT-PCR and amplified with a lowcycle. Then the PCR products were size selected by PAGE gel according to instructions of NEBNext® Multiplex Small RNA Library Prep Set for Illumina® (Illumina, USA). The purified library products were evaluated using the Agilent 2200 TapeStation and the libraries were sequenced by HiSeq 2500 (Illumina, USA) with single-end 50 bp.

### Dual-luciferase reporter assay

We designed the 3’UTR region of ATF3 targeted by miR-760 or mutant target region from Sangon (Shanghai, China), which was inserted into the pmirGLO Dual-Luciferase miRNA Target Expression Vector (Promega), between SacI and SalI sites followed by DNA sequencing confirmation. Cells were seeded in 96-well plates and co-transfected with miR-760 or siMETTL1 and reporter pmirGLO. Dual-Luciferase Reporter Assay System (Promega) was used to evaluate the relative luciferase activity at 48 h after transfection.

### Animal experiments

UM-UC-3 cells (2 × 10^6^ cells per mouse) with stably METTL1 depletion (shMETTL1) were injected into the flanks of mice for the subcutaneous implantation model and the caudal vein for the experimental metastasis model. The tumor growth and tumor metastasis were detected by IVIS Spectrum animal imaging system (PerkinElmer) with 200 μL XenoLight D-luciferin Potassium Salt (15 mg/mL, PerkinElmer) per mouse. The metastases were analyzed by H&E staining. All animals were manipulated according to institutional guidelines and the permission granted by Sir Run Run Shaw Hospital, Zhejiang University.

### Databases and online tools

In this study, we used several databases and online tools to help with the presentation of the results. TCGA database (https://portal.gdc.cancer.gov); Firebrowse (http://firebrowse.org/); Human Protein Atlas (https://www.proteinatlas.org/); LinkedOmics online database (http://www.linkedomics.org/); Oncomine (https://www.oncomine.org/resource/login.html); Volcano plot, Heatmap and Venn diagram were drawn by OmicStudio tools (https://www.omicstudio.cn/tool); Targetscan (http://www.targetscan.org/vert_72/); miRWalk (http://mirwalk.umm.uni-heidelberg.de/); miRDB (http://mirdb.org/); Human Cancer Metastasis Database (http://hcmdb.i-sanger.com/index).

### Statistical analysis

Statistics in this study are displayed as the mean ± SD in GraphPad prism. The statistical distinction between the two groups was estimated by a two-tailed Student’s *t* test. Statistical significance was defined as *P value of <0.05, **P value of <0.01, ***P value of <0.001.

## Supplementary information

Agreement for Authorship change

Western blot original data

Supplementary Figure Legends

Figure Supplementary 1

Figure Supplementary 2

Figure Supplementary 3

Figure Supplementary 4

Figure Supplementary 5

Figure Supplementary 6

Figure Supplementary 7

Table S1

Table S2

Table S3

Table S4

## Data Availability

All data generated or analyzed during this study are included in this published article and its supplementary information files. Further inquiries can be directed to the corresponding author.
