# Supplementary material for: METTL1 drives tumor progression of bladder cancer via degrading ATF3 mRNA in an m7G-modified miR-760-dependent manner
Source: Cell Death Discov. 2022 Nov 17;8:458. doi: 10.1038/s41420-022-01236-6 (PMC9672058; doi:10.1038/s41420-022-01236-6)
Supplement: Supplementary file 1 — Agreement for Authorship change [file 41420_2022_1236_MOESM1_ESM.pdf]

Subject:	Re: Permission for changing the authorship	
From:	3193119@zju.edu.cn (Gonghui Li)	Oct 17, 2022 11:08:53 PM
To:	"谢海云" <12018518@zju.edu.cn>	

I agree.

-----原始邮件-----
发件人:"谢海云" <12018518@zju.edu.cn>
发送时间:2022-10-17 23:04:49 (星期一)
收件人: 3193119@zju.edu.cn, xialiqun@zju.edu.cn, wenjingsu@zju.edu.cn, 21718388@zju.edu.cn, wmc_32@163.com, dinglf@zju.edu.cn, lzy1994@zju.edu.cn, 11818079@zju.edu.cn, 12018327@zju.edu.cn, wenqin.luo@zju.edu.cn, 11818430@zju.edu.cn, 3160102165@zju.edu.cn, 12018518@zju.edu.cn
抄送:
主题: Permission for changing the authorship

Dear Co-authors,

The manuscript (CDDISCOVERY-22-4289) entitled " METTL1 drives tumor progression of bladder cancer via degrading ATF3 mRNA in m7G modified miR-760 dependent manner" were asked to submit a revision. Due to the huge contribution during the revision of this paper, Dr. Haifeng Yu has been listed as the third author. According to the journal's requirements, we are asking all co-authors for agreement to this revision in email. So, if you agree to this change in authorship, please reply "agree".

Thank You!

Sincerely,

Haiyun Xie

Department of Urology,
Sir Run Run Shaw Hospital,
Zhejiang University School of Medicine,

Subject:	Re: Permission for changing the authorship
From:	xialiqun@zju.edu.cn (Liqun Xia) Oct 18, 2022 8:46:43 AM
To:	"谢海云" <12018518@zju.edu.cn>

Agree

-----原始邮件-----
发件人:"谢海云" <12018518@zju.edu.cn>
发送时间:2022-10-17 23:04:49 (星期一)
收件人: 3193119@zju.edu.cn, xialiqun@zju.edu.cn, wenjingsu@zju.edu.cn, 21718388@zju.edu.cn, wmc_32@163.com, dinglf@zju.edu.cn, lzy1994@zju.edu.cn, 11818079@zju.edu.cn, 12018327@zju.edu.cn, wenqin.luo@zju.edu.cn, 11818430@zju.edu.cn, 3160102165@zju.edu.cn, 12018518@zju.edu.cn
抄送:
主题: Permission for changing the authorship

Dear Co-authors,

The manuscript (CDDISCOVERY-22-4289) entitled " METTL1 drives tumor progression of bladder cancer via degrading ATF3 mRNA in m7G modified miR-760 dependent manner" were asked to submit a revision. Due to the huge contribution during the revision of this paper, Dr. Haifeng Yu has been listed as the third author. According to the journal's requirements, we are asking all co-authors for agreement to this revision in email. So, if you agree to this change in authorship, please reply "agree".

Thank You!

Sincerely,

Haiyun Xie

Department of Urology,
Sir Run Run Shaw Hospital,
Zhejiang University School of Medicine,

Subject:	Re: Permission for changing the authorship
From:	"苏文静" <wenjingsu@zju.edu.cn> (Wenjing Su) Oct 18, 2022 9:32:10 AM
To:	"谢海云" <12018518@zju.edu.cn>

Agree

-----原始邮件-----
发件人:"谢海云" <12018518@zju.edu.cn>
发送时间:2022-10-17 23:04:49 (星期一)
收件人: 3193119@zju.edu.cn, xialiqun@zju.edu.cn, wenjingsu@zju.edu.cn, 21718388@zju.edu.cn, wmc_32@163.com, dinglf@zju.edu.cn, lzy1994@zju.edu.cn, 11818079@zju.edu.cn, 12018327@zju.edu.cn, wenqin.luo@zju.edu.cn, 11818430@zju.edu.cn, 3160102165@zju.edu.cn, 12018518@zju.edu.cn
抄送:
主题: Permission for changing the authorship

Dear Co-authors,

The manuscript (CDDISCOVERY-22-4289) entitled " METTL1 drives tumor progression of bladder cancer via degrading ATF3 mRNA in m7G modified miR-760 dependent manner" were asked to submit a revision. Due to the huge contribution during the revision of this paper, Dr. Haifeng Yu has been listed as the third author. According to the journal's requirements, we are asking all co-authors for agreement to this revision in email. So, if you agree to this change in authorship, please reply "agree".

Thank You!

Sincerely,

Haiyun Xie

Department of Urology,
Sir Run Run Shaw Hospital,
Zhejiang University School of Medicine,

Subject:	Permission for changing the authorship
From:	"哎" <1156959554@qq.com> (wmc_32@163.com Mingchao Wang) Oct 18, 2022 6:29:58 AM
To:	"谢海云" <12018518@zju.edu.cn>

I agree.

---原始邮件---

发件人："谢海云"<12018518@zju.edu.cn>

发送时间："undefined"

收件人："3193119"<3193119@zju.edu.cn>,"xialiqun","wenjingsu","21718388" <21718388@zju.edu.cn>,"wmc_32","dinglf","lzy1994","11818079"<11818079@zju.edu.cn>,"12018327" <12018327@zju.edu.cn>,"wenqin.luo","11818430"<11818430@zju.edu.cn>,"3160102165"<3160102165@zju.edu.cn>,"12018518" <12018518@zju.edu.cn>

主题：Permission for changing the authorship

Dear Co-authors,

The manuscript (CDDISCOVERY-22-4289) entitled " METTL1 drives tumor progression of bladder cancer via degrading ATF3 mRNA in m7G modified miR-760 dependent manner" were asked to submit a revision. Due to the huge contribution during the revision of this paper, Dr. Haifeng Yu has been listed as the third author. According to the journal's requirements, we are asking all co-authors for agreement to this revision in email. So, if you agree to this change in authorship, please reply "agree".

Thank You!

Sincerely,

Haiyun Xie

Department of Urology,
Sir Run Run Shaw Hospital,
Zhejiang University School of Medicine,

Subject:	回复: Permission for changing the authorship
From:	wanghuan <21718388@zju.edu.cn> (Huan Wang) Oct 18, 2022 5:10:37 PM
To:	"谢海云" <12018518@zju.edu.cn>

Agree

从 Windows 版邮件发送

发件人: 谢海云
发送时间: 2022年10月17日 23:04
收件人: 3193119@zju.edu.cn; xialiqun@zju.edu.cn; wenjingsu@zju.edu.cn; 21718388@zju.edu.cn; wmc_32@163.com; dinglf@zju.edu.cn; lzy1994@zju.edu.cn; 11818079@zju.edu.cn; 12018327@zju.edu.cn; wenqin.luo@zju.edu.cn; 11818430@zju.edu.cn; 3160102165@zju.edu.cn; 12018518@zju.edu.cn
主题: Permission for changing the authorship

Dear Co-authors,

The manuscript (CDDISCOVERY-22-4289) entitled " METTL1 drives tumor progression of bladder cancer via degrading ATF3 mRNA in m7G modified miR-760 dependent manner" were asked to submit a revision. Due to the huge contribution during the revision of this paper, Dr. Haifeng Yu has been listed as the third author. According to the journal's requirements, we are asking all co-authors for agreement to this revision in email. So, if you agree to this change in authorship, please reply "agree".

Thank You!

Sincerely,

Haiyun Xie

Department of Urology,

Sir Run Run Shaw Hospital,

Zhejiang University School of Medicine,

Subject:	Re: Permission for changing the authorship
From:	"丁理峰" <dinglf@zju.edu.cn> (Lifeng Ding) Oct 18, 2022 4:51:12 PM
To:	"谢海云" <12018518@zju.edu.cn>

I agree.

-----原始邮件-----
发件人:"谢海云" <12018518@zju.edu.cn>
发送时间:2022-10-17 23:04:49 (星期一)
收件人: 3193119@zju.edu.cn, xialiqun@zju.edu.cn, wenjingsu@zju.edu.cn, 21718388@zju.edu.cn, wmc_32@163.com, dinglf@zju.edu.cn, lzy1994@zju.edu.cn, 11818079@zju.edu.cn, 12018327@zju.edu.cn, wenqin.luo@zju.edu.cn, 11818430@zju.edu.cn, 3160102165@zju.edu.cn, 12018518@zju.edu.cn
抄送:
主题: Permission for changing the authorship

Dear Co-authors,

The manuscript (CDDISCOVERY-22-4289) entitled " METTL1 drives tumor progression of bladder cancer via degrading ATF3 mRNA in m7G modified miR-760 dependent manner" were asked to submit a revision. Due to the huge contribution during the revision of this paper, Dr. Haifeng Yu has been listed as the third author. According to the journal's requirements, we are asking all co-authors for agreement to this revision in email. So, if you agree to this change in authorship, please reply "agree".

Thank You!

Sincerely,

Haiyun Xie

Department of Urology,
Sir Run Run Shaw Hospital,
Zhejiang University School of Medicine,

Subject:	Re: Permission for changing the authorship	
From:	"路边" <harrylu1994@foxmail.com> (lzy1994@zju.edu.cn Zeyi Lu)	Oct 17, 2022 11:25:24 PM
To:	"谢海云" <12018518@zju.edu.cn>	

Agree

发自我的iPhone

------------------ Original ------------------

From: 谢海云 <12018518@zju.edu.cn>
Date: Mon,Oct 17,2022 11:04 PM
To: 3193119 <3193119@zju.edu.cn>, xialiqun <xialiqun@zju.edu.cn>, wenjingsu <wenjingsu@zju.edu.cn>, 21718388 <21718388@zju.edu.cn>, wmc_32 <wmc_32@163.com>, dinglf <dinglf@zju.edu.cn>, lzy1994 <lzy1994@zju.edu.cn>, 11818079 <11818079@zju.edu.cn>, 12018327 <12018327@zju.edu.cn>, wenqin.luo <wenqin.luo@zju.edu.cn>, 11818430 <11818430@zju.edu.cn>, 3160102165 <3160102165@zju.edu.cn>, 12018518 <12018518@zju.edu.cn>
Subject: Re: Permission for changing the authorship

Dear Co-authors,

The manuscript (CDDISCOVERY-22-4289) entitled " METTL1 drives tumor progression of bladder cancer via degrading ATF3 mRNA in m7G modified miR-760 dependent manner" were asked to submit a revision. Due to the huge contribution during the revision of this paper, Dr. Haifeng Yu has been listed as the third author. According to the journal's requirements, we are asking all co-authors for agreement to this revision in email. So, if you agree to this change in authorship, please reply "agree".

Thank You!

Sincerely,

Haiyun Xie

Department of Urology,
Sir Run Run Shaw Hospital,
Zhejiang University School of Medicine,

Subject:	Re: Permission for changing the authorship
From:	"郑起明" <11818079@zju.edu.cn> (Qiming Zheng) Oct 18, 2022 12:13:05 PM
To:	"谢海云" <12018518@zju.edu.cn>

I agree.

-----原始邮件-----
发件人:"谢海云" <12018518@zju.edu.cn>
发送时间:2022-10-17 23:04:49 (星期一)
收件人: 3193119@zju.edu.cn, xialiqun@zju.edu.cn, wenjingsu@zju.edu.cn, 21718388@zju.edu.cn, wmc_32@163.com, dinglf@zju.edu.cn, lzy1994@zju.edu.cn, 11818079@zju.edu.cn, 12018327@zju.edu.cn, wenqin.luo@zju.edu.cn, 11818430@zju.edu.cn, 3160102165@zju.edu.cn, 12018518@zju.edu.cn
抄送:
主题: Permission for changing the authorship

Dear Co-authors,

The manuscript (CDDISCOVERY-22-4289) entitled " METTL1 drives tumor progression of bladder cancer via degrading ATF3 mRNA in m7G modified miR-760 dependent manner" were asked to submit a revision. Due to the huge contribution during the revision of this paper, Dr. Haifeng Yu has been listed as the third author. According to the journal's requirements, we are asking all co-authors for agreement to this revision in email. So, if you agree to this change in authorship, please reply "agree".

Thank You!

Sincerely,

Haiyun Xie

Department of Urology,
Sir Run Run Shaw Hospital,
Zhejiang University School of Medicine,

Subject:	Re: Permission for changing the authorship
From:	"王茹玥" <12018327@zju.edu.cn> (Ruyue Wang) Oct 18, 2022 4:46:00 PM
To:	"谢海云" <12018518@zju.edu.cn>

agree

-----原始邮件-----
发件人:"谢海云" <12018518@zju.edu.cn>
发送时间:2022-10-17 23:04:49 (星期一)
收件人: 3193119@zju.edu.cn, xialiqun@zju.edu.cn, wenjingsu@zju.edu.cn, 21718388@zju.edu.cn, wmc_32@163.com, dinglf@zju.edu.cn, lzy1994@zju.edu.cn, 11818079@zju.edu.cn, 12018327@zju.edu.cn, wenqin.luo@zju.edu.cn, 11818430@zju.edu.cn, 3160102165@zju.edu.cn, 12018518@zju.edu.cn
抄送:
主题: Permission for changing the authorship

Dear Co-authors,

The manuscript (CDDISCOVERY-22-4289) entitled " METTL1 drives tumor progression of bladder cancer via degrading ATF3 mRNA in m7G modified miR-760 dependent manner" were asked to submit a revision. Due to the huge contribution during the revision of this paper, Dr. Haifeng Yu has been listed as the third author. According to the journal's requirements, we are asking all co-authors for agreement to this revision in email. So, if you agree to this change in authorship, please reply "agree".

Thank You!

Sincerely,

Haiyun Xie

Department of Urology,
Sir Run Run Shaw Hospital,
Zhejiang University School of Medicine,

Subject:	回复：Permission for changing the authorship	
From:	"12018647@zju.edu.cn" <12018647@zju.edu.cn> (wenqin.luo@zju.edu.cn Wenqin Luo)	Oct 17, 2022 11:12:52 PM
To:	"谢海云" <12018518@zju.edu.cn>	

I agree.

发自我的华为手机

-------- 原始邮件 --------
主题：Permission for changing the authorship
发件人：谢海云 <12018518@zju.edu.cn>
收件人：
3193119@zju.edu.cn,xialiqun@zju.edu.cn,wenjingsu@zju.edu.cn,21718388@zju.edu.cn,wmc_32@163.com,dinglf@zju.edu.cn,lzy1994@zju.edu.cn,11818079@zju.edu.cn,12018327@zju.edu.cn,wenqin.l
抄送：

> Dear Co-authors,
>
> The manuscript (CDDISCOVERY-22-4289) entitled " METTL1 drives tumor progression of bladder cancer via degrading ATF3 mRNA in m7G modified miR-760 dependent manner" were asked to submit a revision. Due to the huge contribution during the revision of this paper, Dr. Haifeng Yu has been listed as the third author. According to the journal's requirements, we are asking all co-authors for agreement to this revision in email. So, if you agree to this change in authorship, please reply "agree".
>
> Thank You!
>
> Sincerely,
>
> Haiyun Xie
>
> Department of Urology,
> Sir Run Run Shaw Hospital,
> Zhejiang University School of Medicine,

Subject:	Re: Permission for changing the authorship
From:	"任亮亮" <11818430@zju.edu.cn> (Liangliang Ren) Oct 18, 2022 8:48:50 PM
To:	"谢海云" <12018518@zju.edu.cn>
Cc:	3193119@zju.edu.cn, xialiqun@zju.edu.cn, wenjingsu@zju.edu.cn, 21718388@zju.edu.cn, wmc_32@163.com, dinglf@zju.edu.cn, lzy1994@zju.edu.cn, 11818079@zju.edu.cn, 12018327@zju.edu.cn, wenqin.luo@zju.edu.cn, 3160102165@zju.edu.cn

I agree.

2022-10-17 23:04:49"谢海云" <12018518@zju.edu.cn>写道:

> Dear Co-authors,
>
> The manuscript (CDDISCOVERY-22-4289) entitled " METTL1 drives tumor progression of bladder cancer via degrading ATF3 mRNA in m7G modified miR-760 dependent manner" were asked to submit a revision. Due to the huge contribution during the revision of this paper, Dr. Haifeng Yu has been listed as the third author. According to the journal's requirements, we are asking all co-authors for agreement to this revision in email. So, if you agree to this change in authorship, please reply "agree".
>
> Thank You!
>
> Sincerely,
>
> Haiyun Xie
>
> Department of Urology,
> Sir Run Run Shaw Hospital,
> Zhejiang University School of Medicine,

Subject:	Re: Permission for changing the authorship
From:	"周震威" <3160102165@zju.edu.cn> (Zhenwei Zhou) Oct 18, 2022 5:04:12 PM
To:	"谢海云" <12018518@zju.edu.cn>

I agree

-----原始邮件-----
发件人:"谢海云" <12018518@zju.edu.cn>
发送时间:2022-10-17 23:04:49 (星期一)
收件人: 3193119@zju.edu.cn, xialiqun@zju.edu.cn, wenjingsu@zju.edu.cn, 21718388@zju.edu.cn, wmc_32@163.com, dinglf@zju.edu.cn, lzy1994@zju.edu.cn, 11818079@zju.edu.cn, 12018327@zju.edu.cn, wenqin.luo@zju.edu.cn, 11818430@zju.edu.cn, 3160102165@zju.edu.cn, 12018518@zju.edu.cn
抄送:
主题: Permission for changing the authorship

Dear Co-authors,

The manuscript (CDDISCOVERY-22-4289) entitled " METTL1 drives tumor progression of bladder cancer via degrading ATF3 mRNA in m7G modified miR-760 dependent manner" were asked to submit a revision. Due to the huge contribution during the revision of this paper, Dr. Haifeng Yu has been listed as the third author. According to the journal's requirements, we are asking all co-authors for agreement to this revision in email. So, if you agree to this change in authorship, please reply "agree".

Thank You!

Sincerely,

Haiyun Xie

Department of Urology,
Sir Run Run Shaw Hospital,
Zhejiang University School of Medicine,

Zhenwei Zhou, Ba Denian Medicine Class,
Chu Kochen Honors College | College of life science Zhejiang University
Tel: (+86)188-6811-2569
E-mail: zhenweizhou@zju.edu.cn
