# Supplementary material for: METTL1 drives tumor progression of bladder cancer via degrading ATF3 mRNA in an m7G-modified miR-760-dependent manner
Source: Cell Death Discov. 2022 Nov 17;8:458. doi: 10.1038/s41420-022-01236-6 (PMC9672058; doi:10.1038/s41420-022-01236-6)
Supplement: Supplementary file 2 — Western blot original data [file 41420_2022_1236_MOESM2_ESM.pptx]

## Slide 1
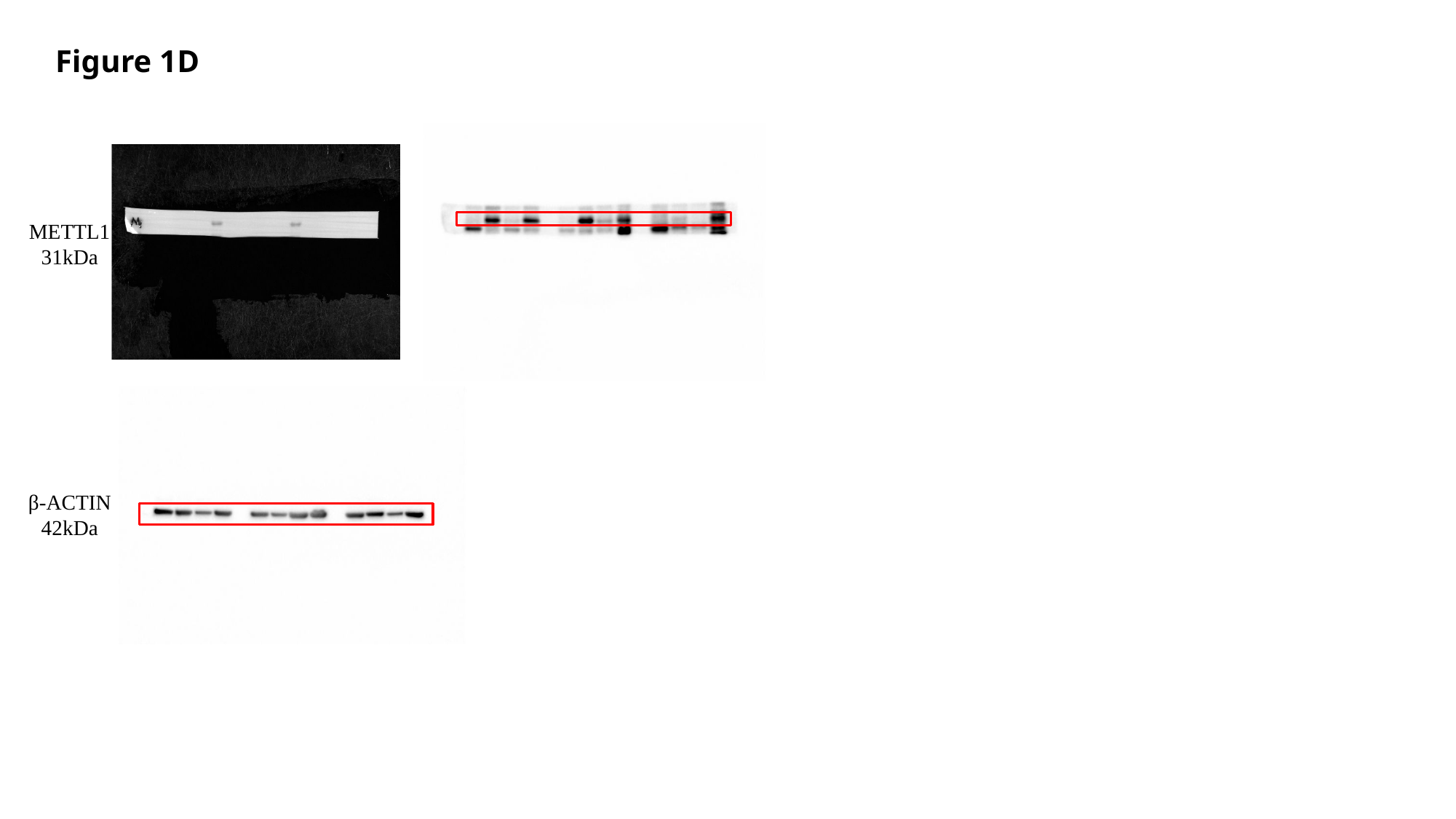

Figure 1D
METTL1
31kDa
β-ACTIN
42kDa

## Slide 2
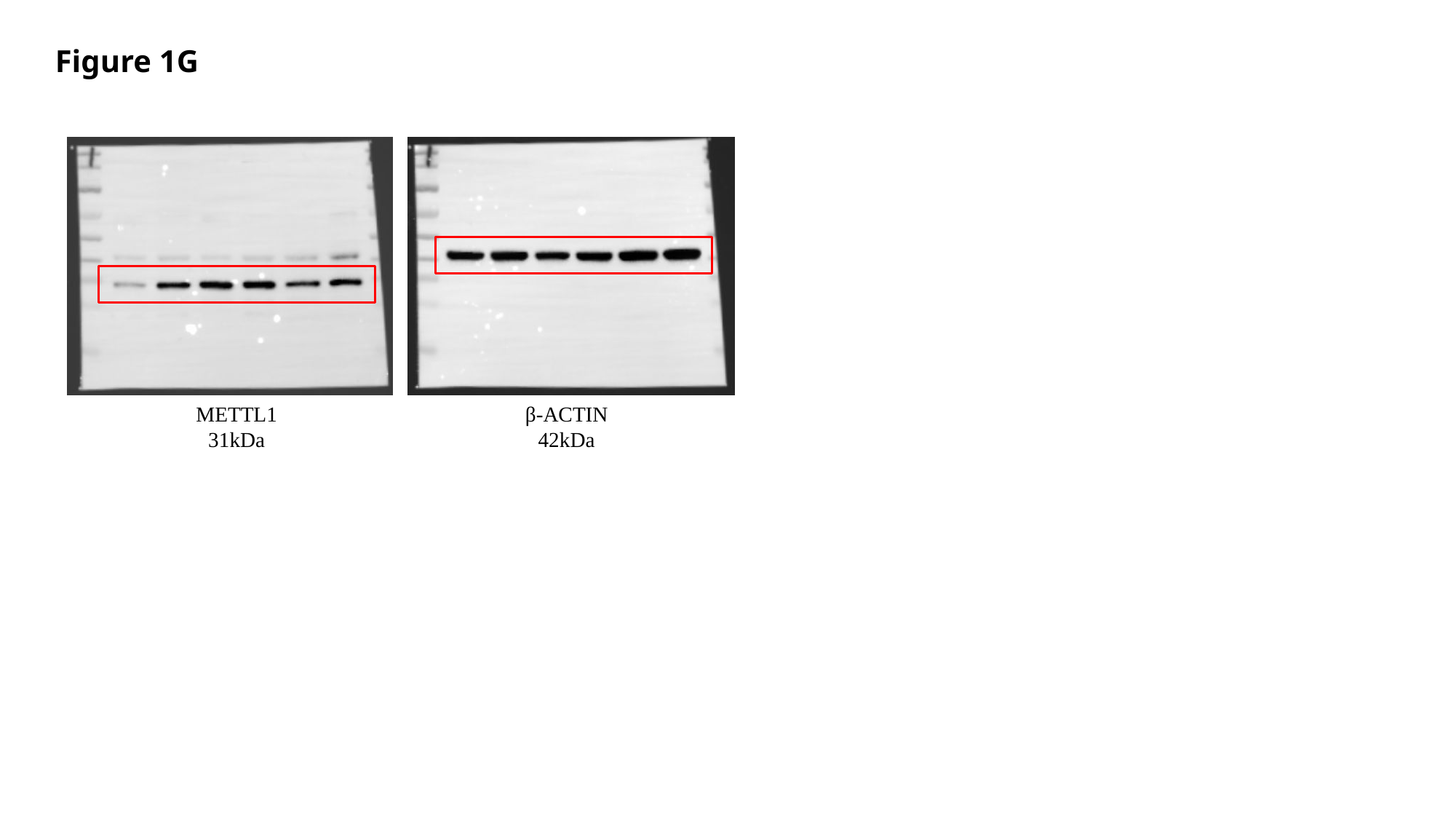

Figure 1G
METTL1
31kDa
β-ACTIN
42kDa

## Slide 3
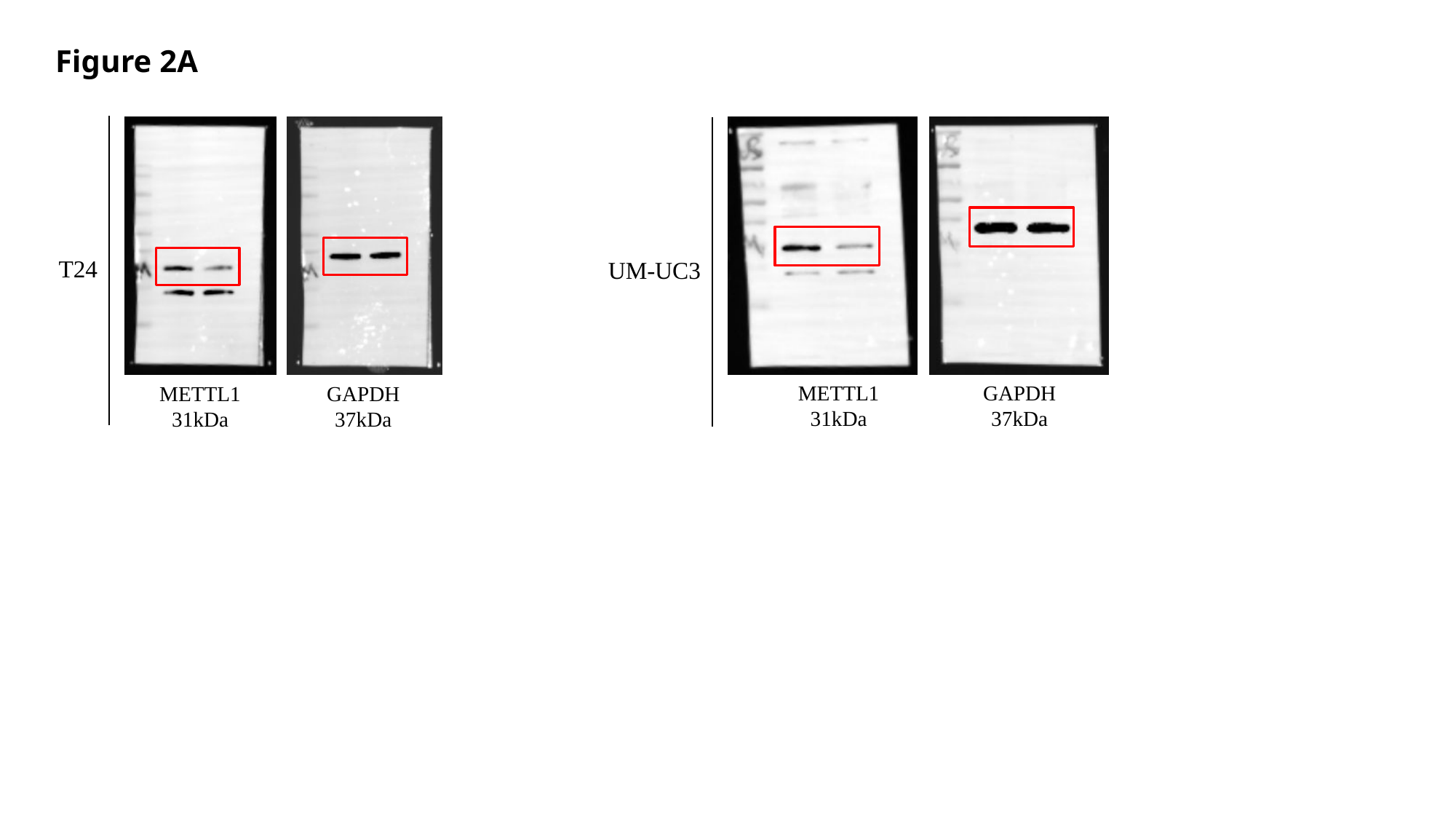

Figure 2A
T24
UM-UC3
METTL1
31kDa
GAPDH
37kDa
METTL1
31kDa
GAPDH
37kDa

## Slide 4
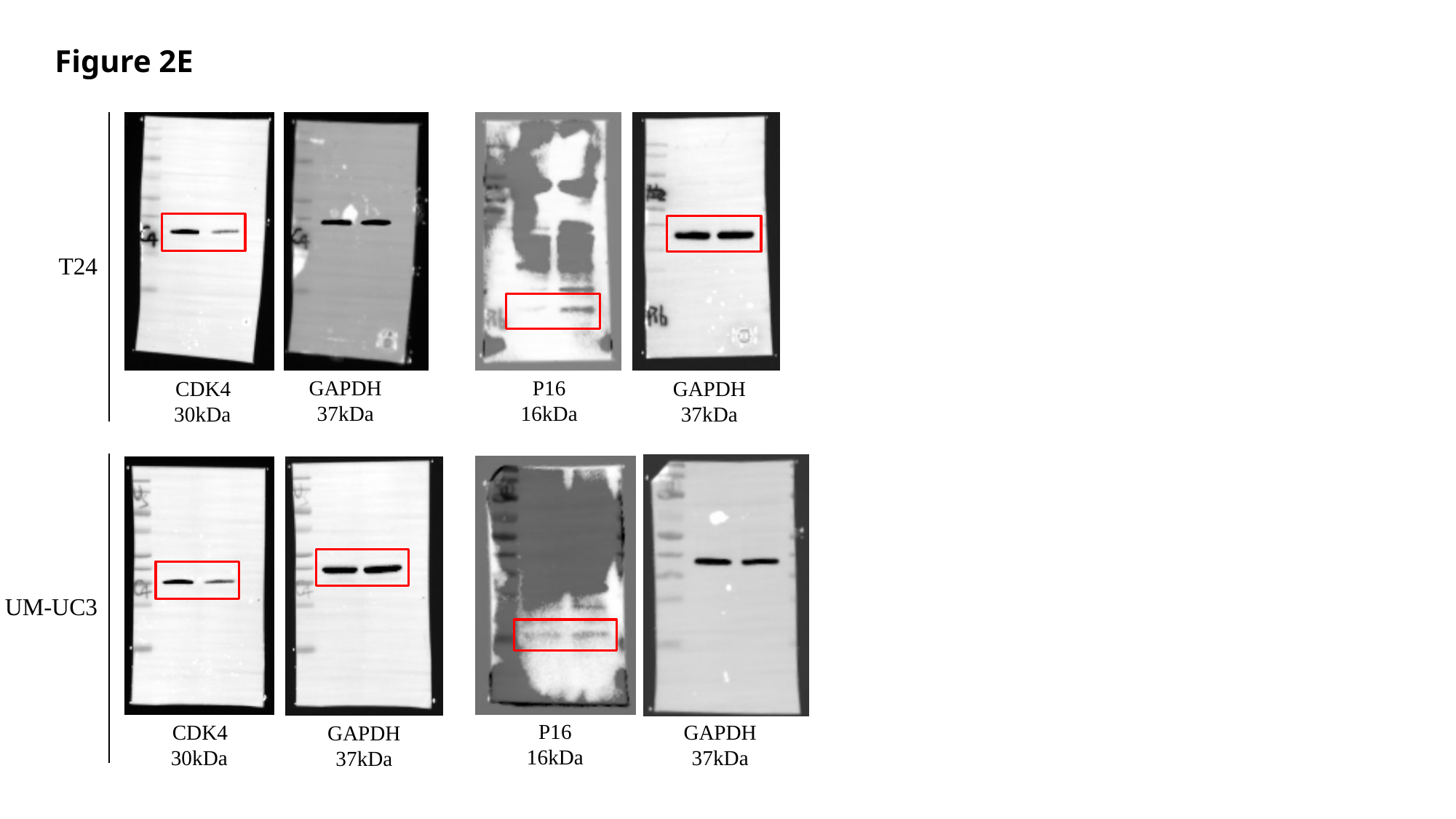

Figure 2E
T24
P16
16kDa
GAPDH
37kDa
CDK4
30kDa
GAPDH
37kDa
UM-UC3
P16
16kDa
CDK4
30kDa
GAPDH
37kDa
GAPDH
37kDa

## Slide 5
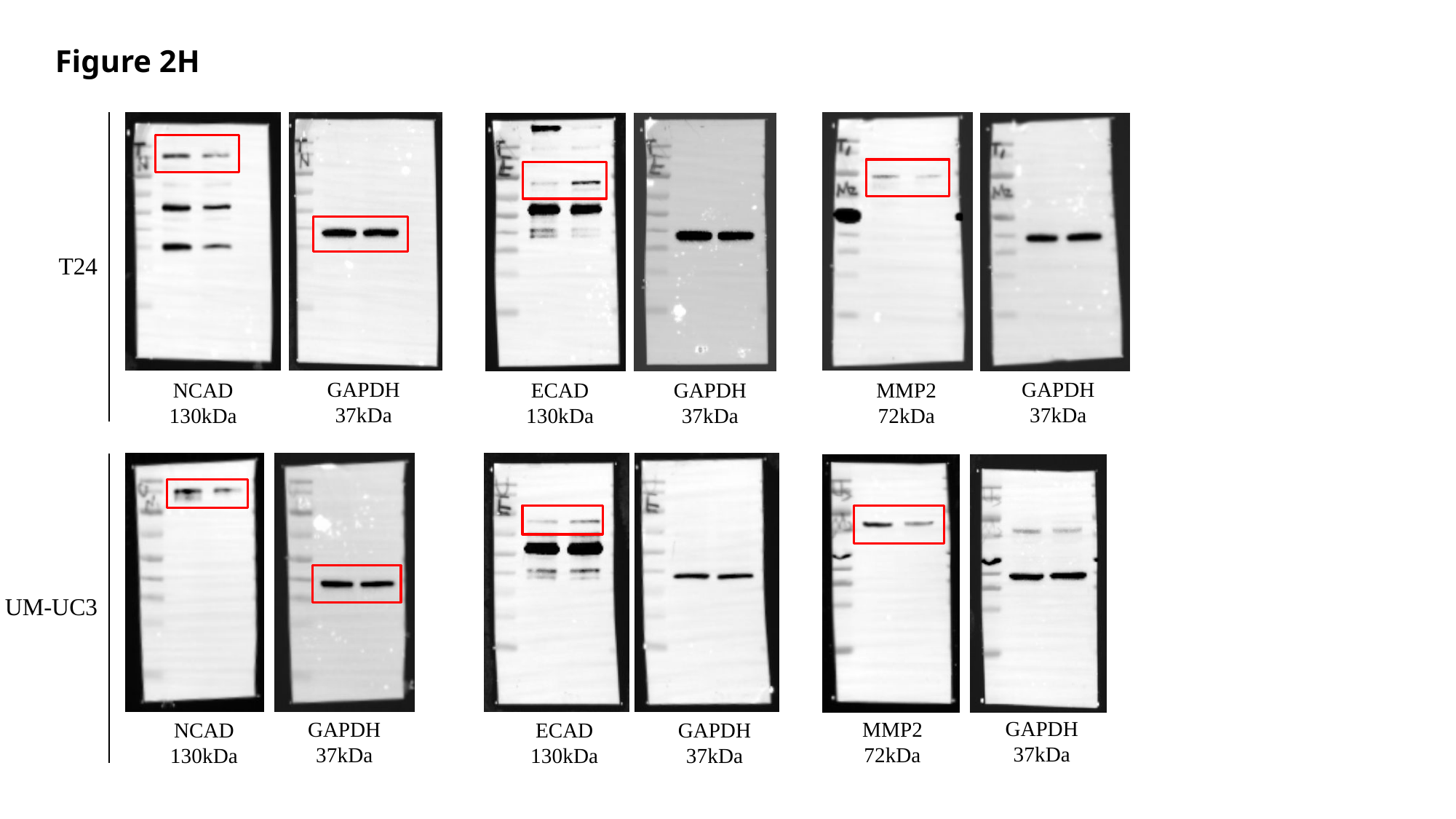

Figure 2H
T24
GAPDH
37kDa
GAPDH
37kDa
NCAD
130kDa
ECAD
130kDa
GAPDH
37kDa
MMP2
72kDa
UM-UC3
GAPDH
37kDa
GAPDH
37kDa
MMP2
72kDa
NCAD
130kDa
ECAD
130kDa
GAPDH
37kDa

## Slide 6
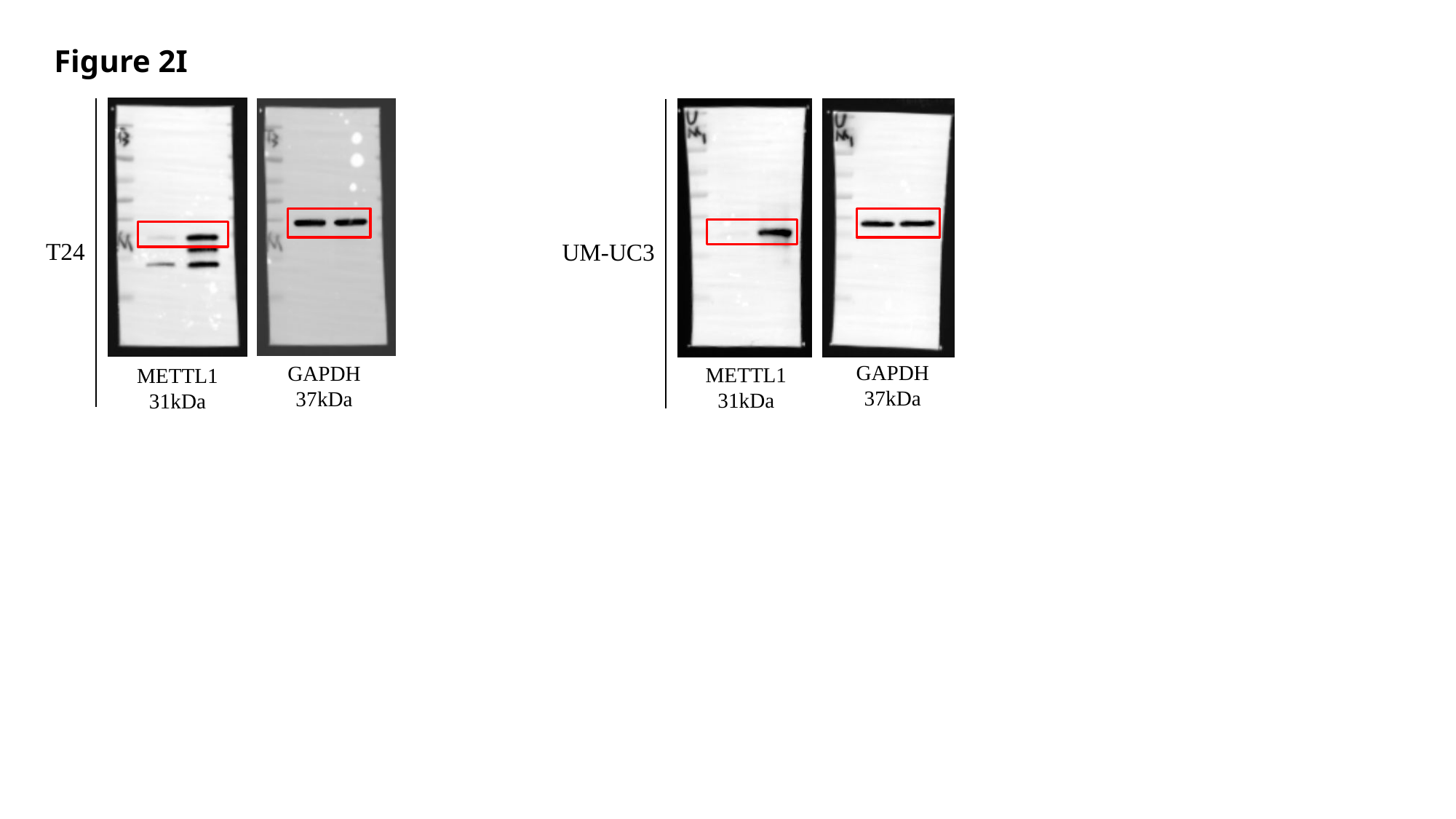

Figure 2I
T24
UM-UC3
GAPDH
37kDa
GAPDH
37kDa
METTL1
31kDa
METTL1
31kDa

## Slide 7
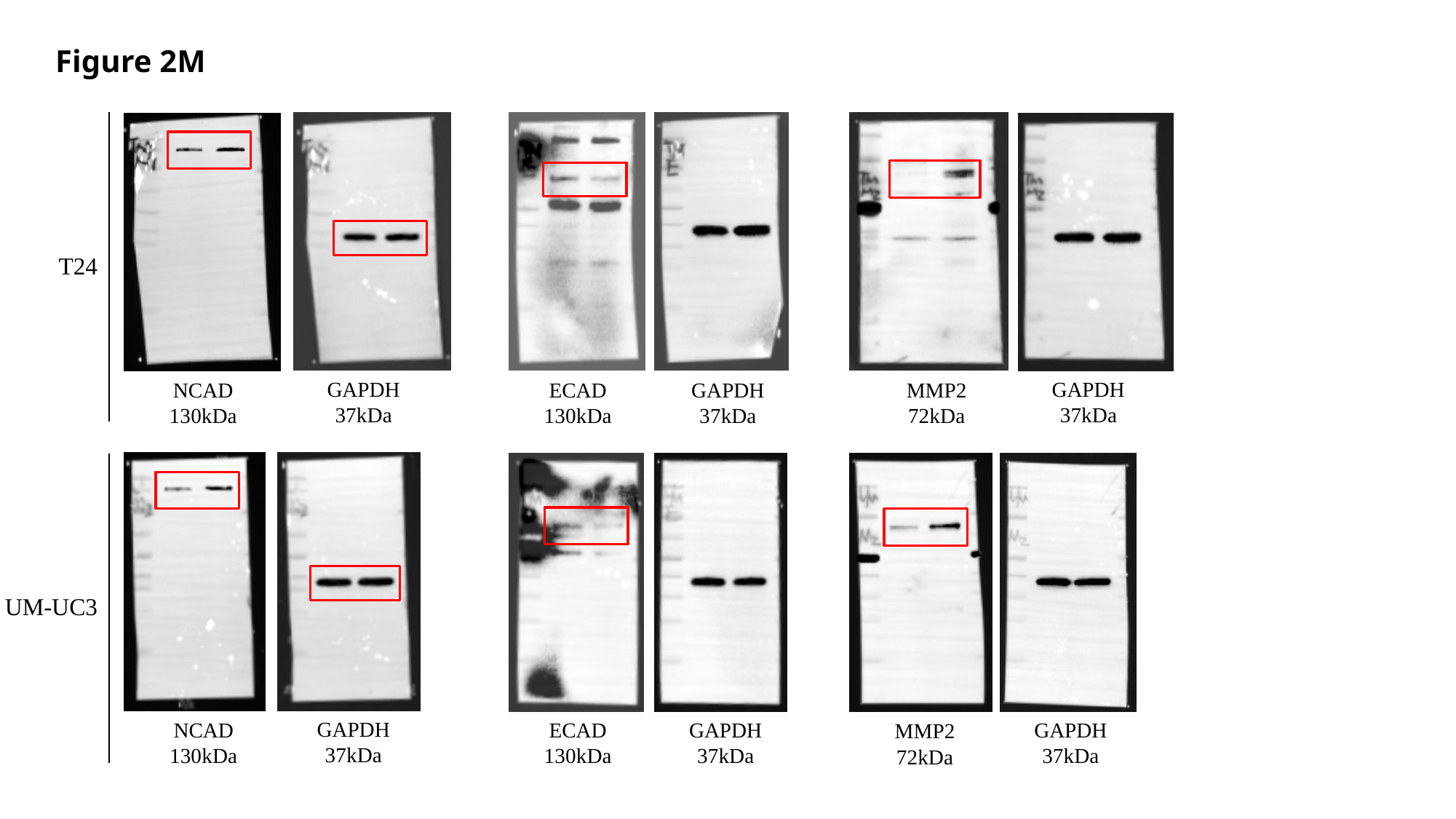

Figure 2M
T24
GAPDH
37kDa
GAPDH
37kDa
NCAD
130kDa
ECAD
130kDa
GAPDH
37kDa
MMP2
72kDa
UM-UC3
GAPDH
37kDa
ECAD
130kDa
GAPDH
37kDa
GAPDH
37kDa
NCAD
130kDa
MMP2
72kDa

## Slide 8
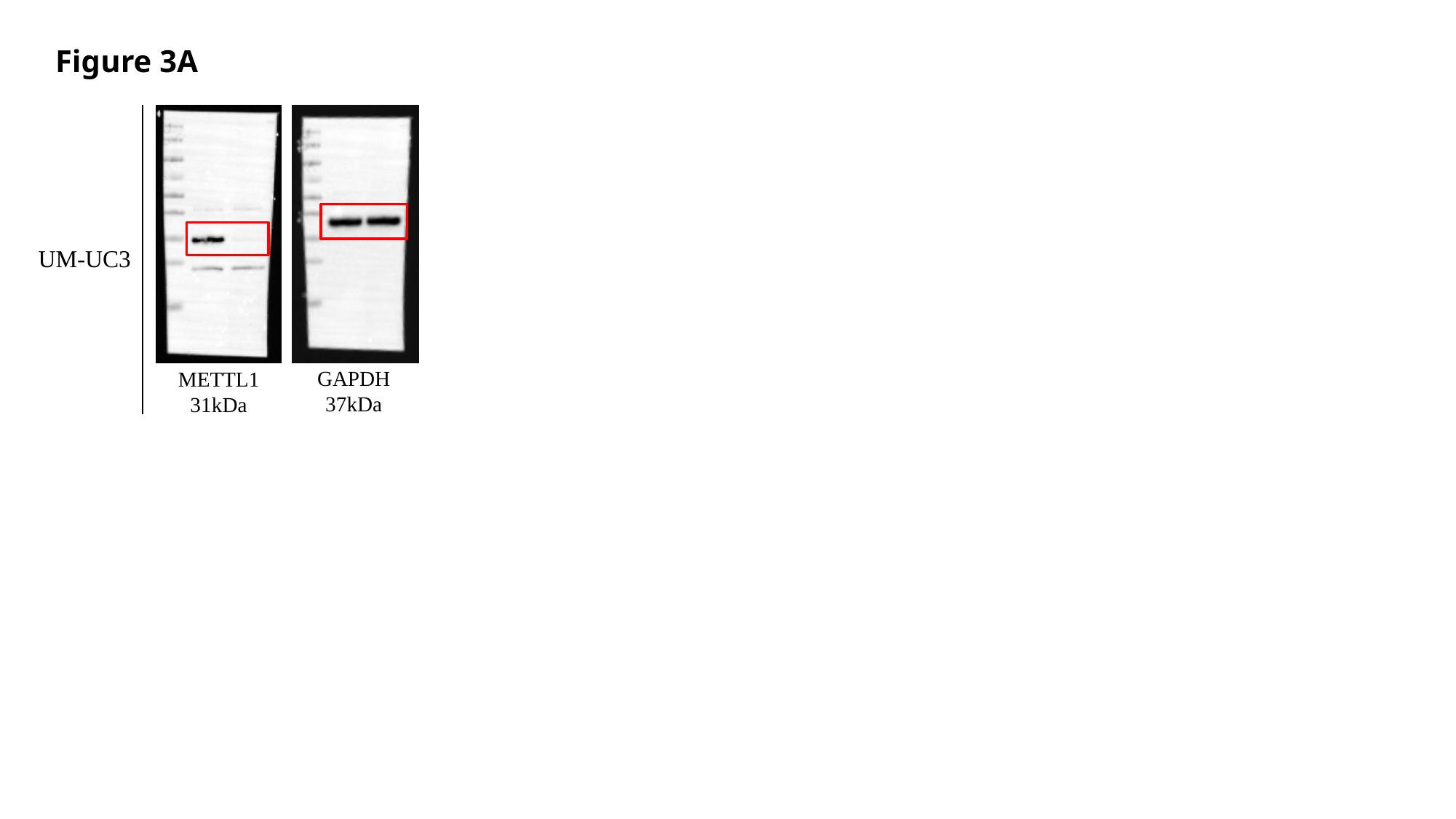

Figure 3A
UM-UC3
GAPDH
37kDa
METTL1
31kDa

## Slide 9
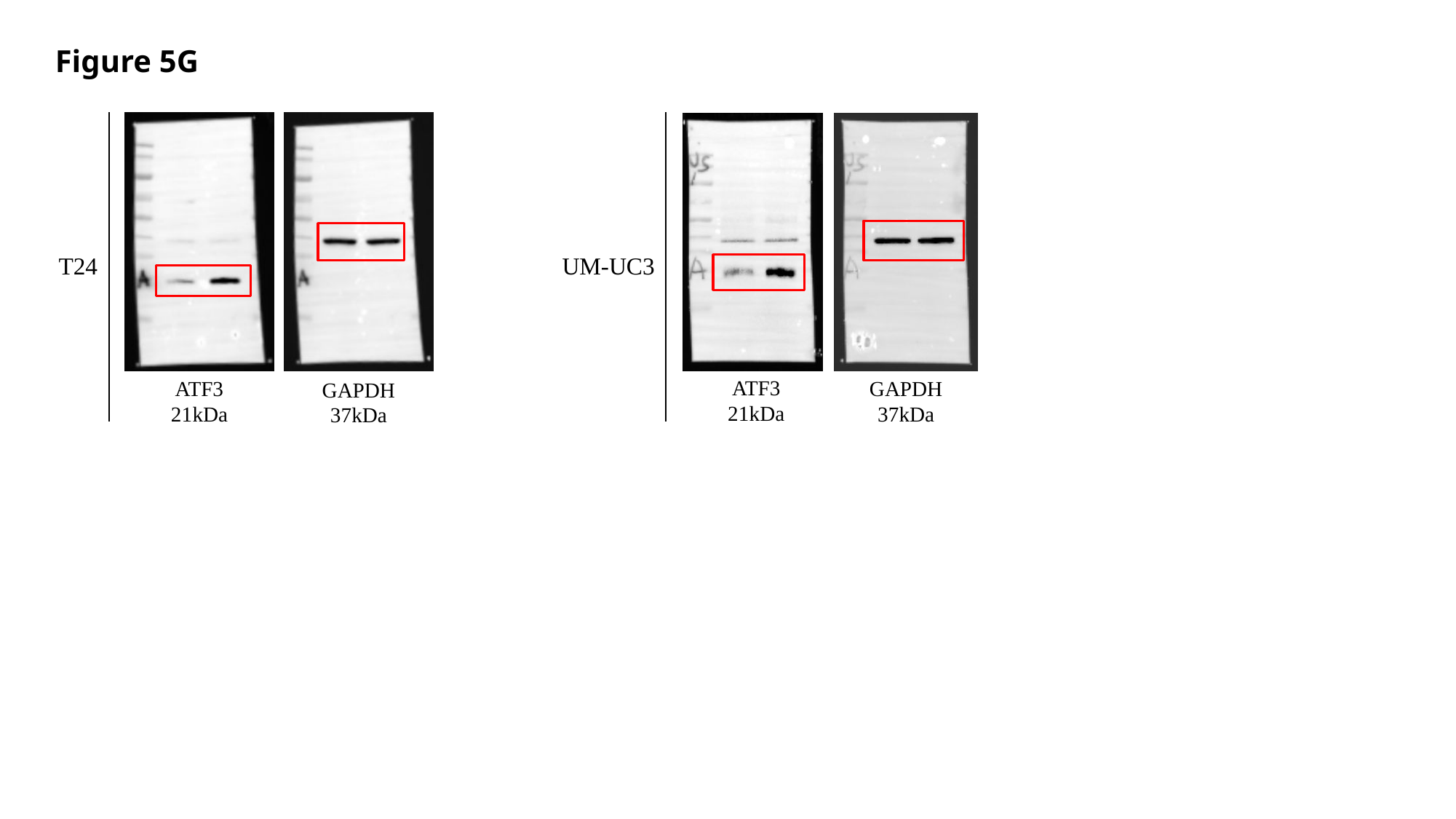

Figure 5G
T24
UM-UC3
ATF3
21kDa
ATF3
21kDa
GAPDH
37kDa
GAPDH
37kDa

## Slide 10
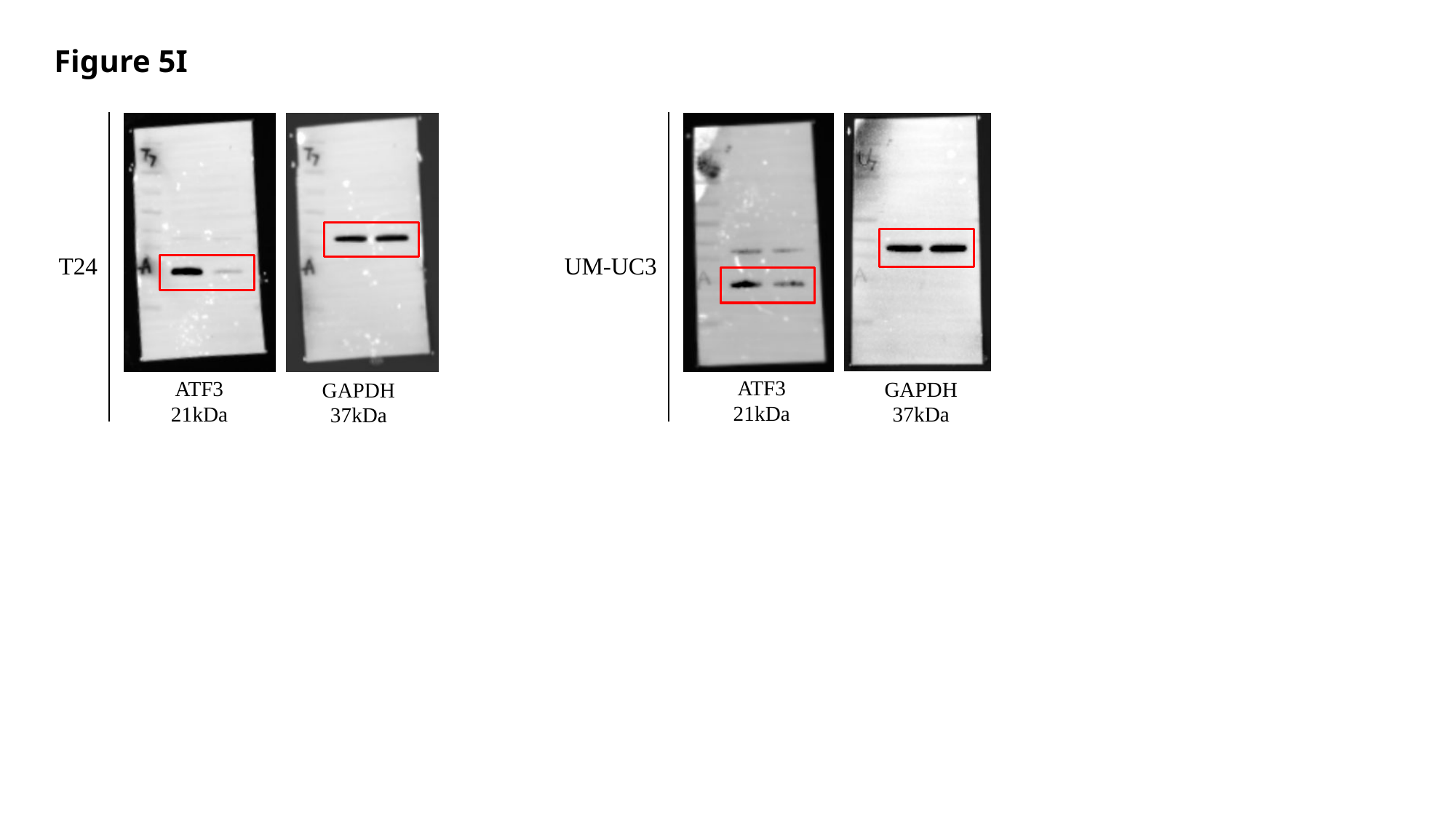

Figure 5I
T24
UM-UC3
ATF3
21kDa
ATF3
21kDa
GAPDH
37kDa
GAPDH
37kDa

## Slide 11
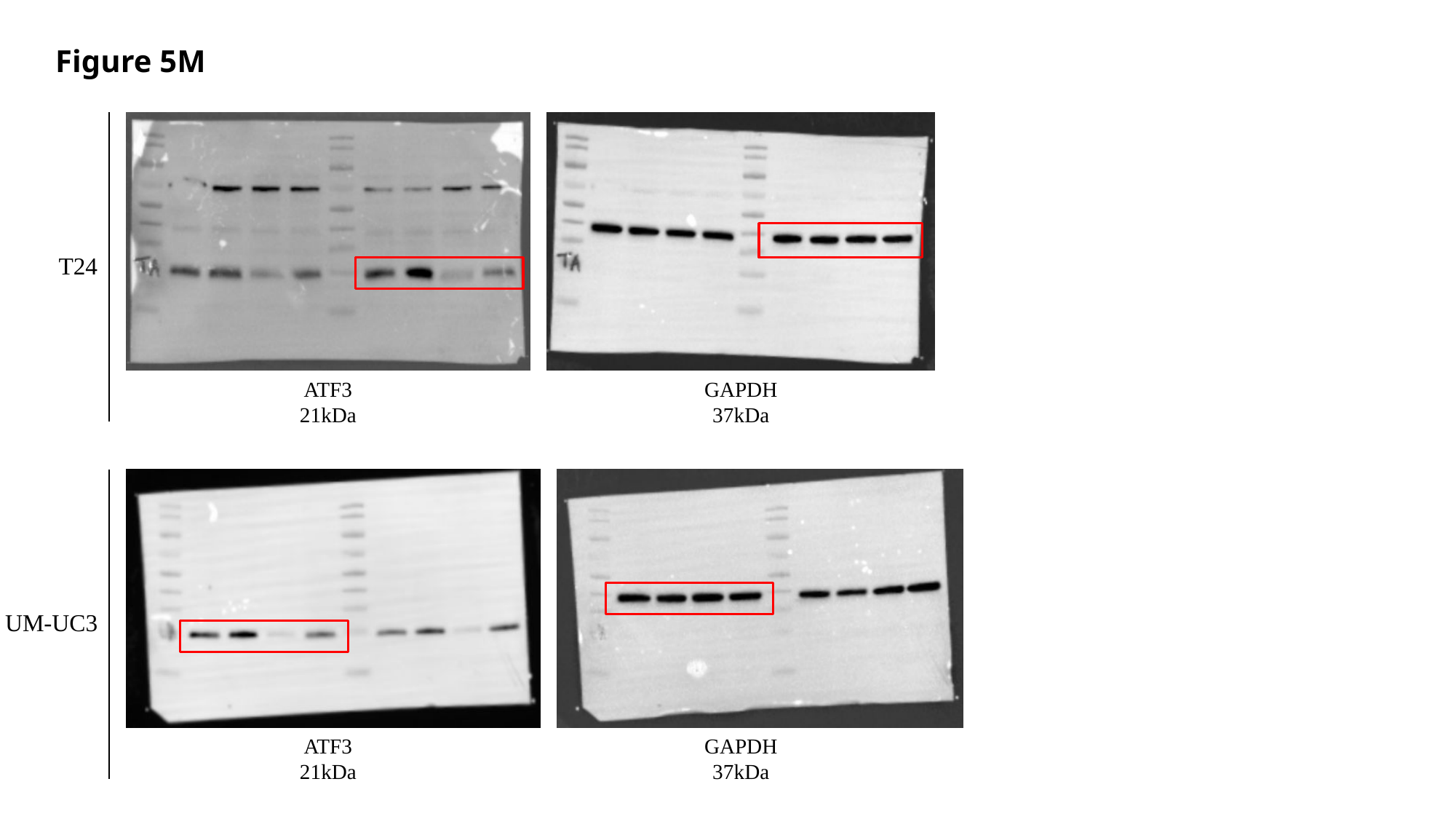

Figure 5M
T24
ATF3
21kDa
GAPDH
37kDa
UM-UC3
ATF3
21kDa
GAPDH
37kDa

## Slide 12
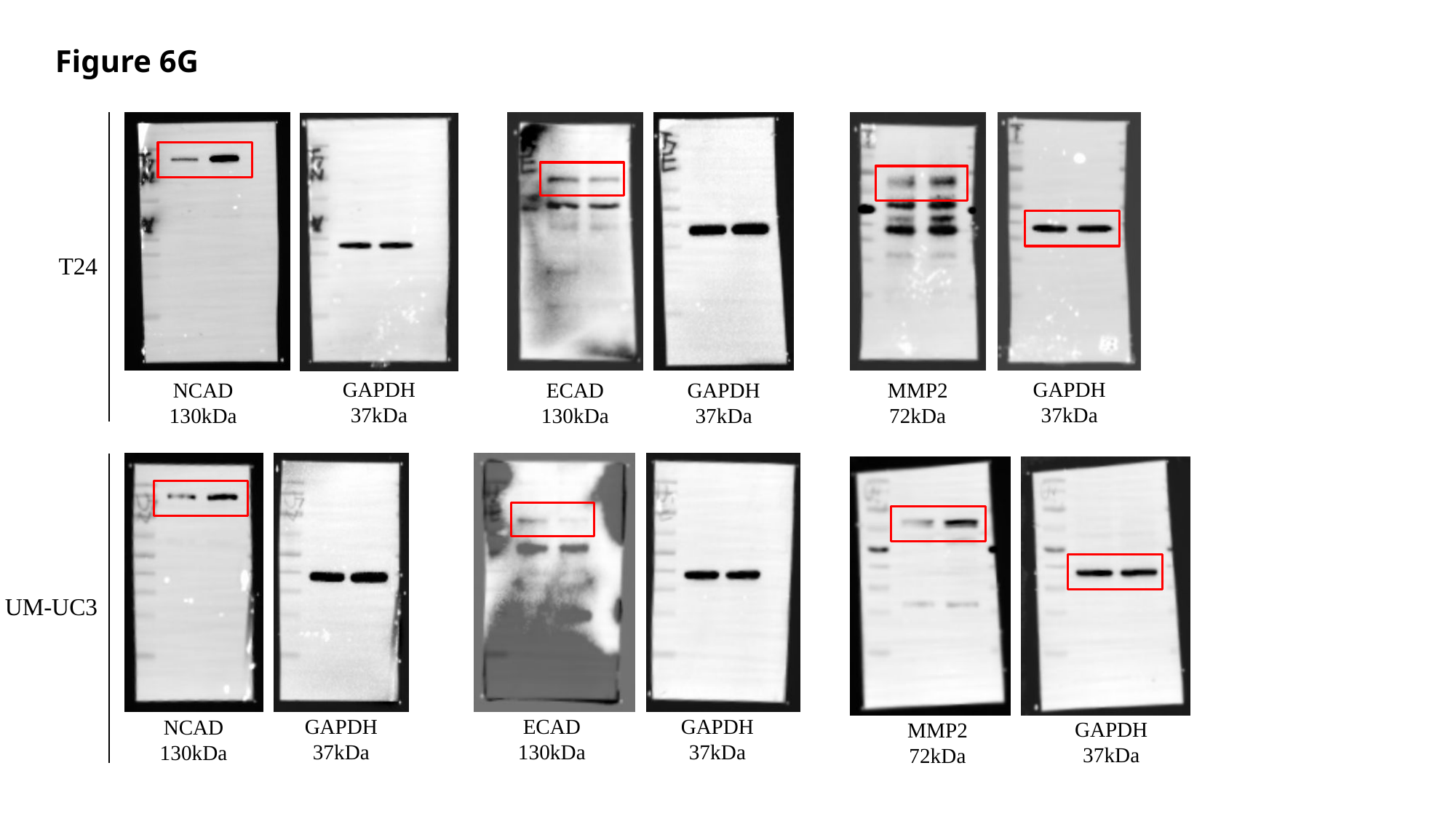

Figure 6G
T24
GAPDH
37kDa
GAPDH
37kDa
NCAD
130kDa
ECAD
130kDa
GAPDH
37kDa
MMP2
72kDa
UM-UC3
ECAD
130kDa
GAPDH
37kDa
GAPDH
37kDa
NCAD
130kDa
GAPDH
37kDa
MMP2
72kDa

## Slide 13
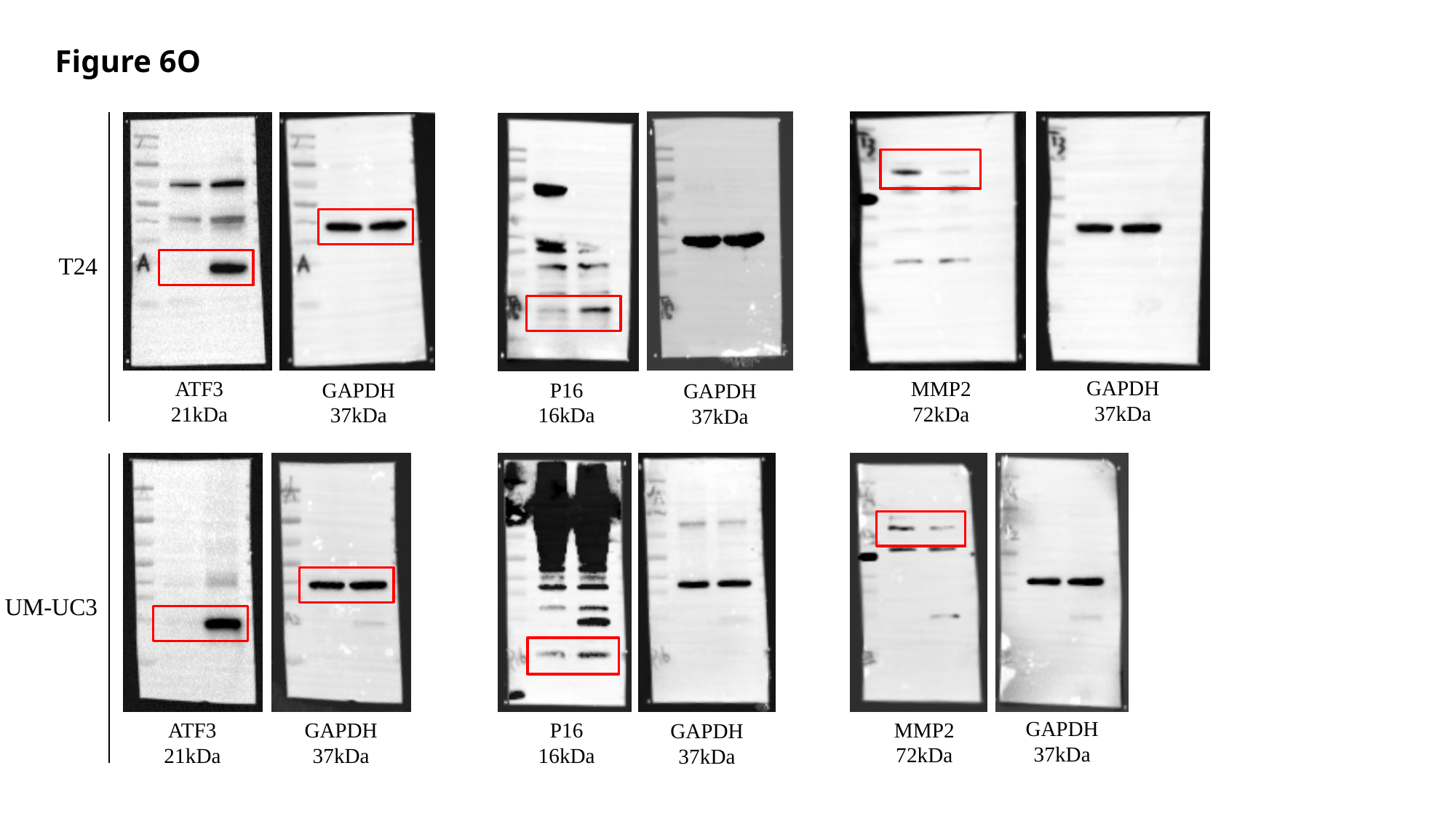

Figure 6O
T24
GAPDH
37kDa
ATF3
21kDa
MMP2
72kDa
GAPDH
37kDa
P16
16kDa
GAPDH
37kDa
UM-UC3
GAPDH
37kDa
MMP2
72kDa
ATF3
21kDa
GAPDH
37kDa
P16
16kDa
GAPDH
37kDa

## Slide 14
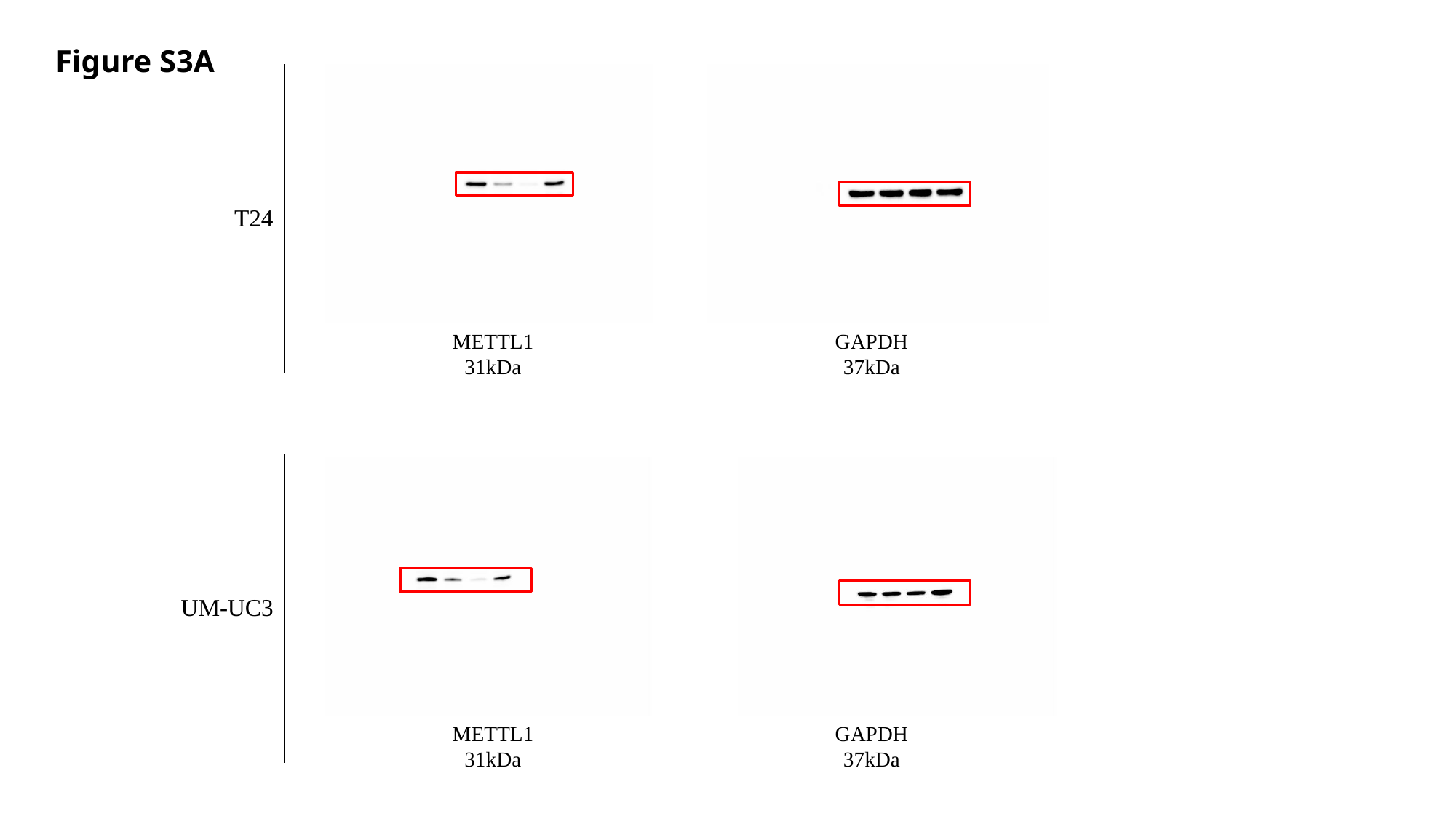

Figure S3A
T24
METTL1
31kDa
GAPDH
37kDa
UM-UC3
METTL1
31kDa
GAPDH
37kDa

## Slide 15
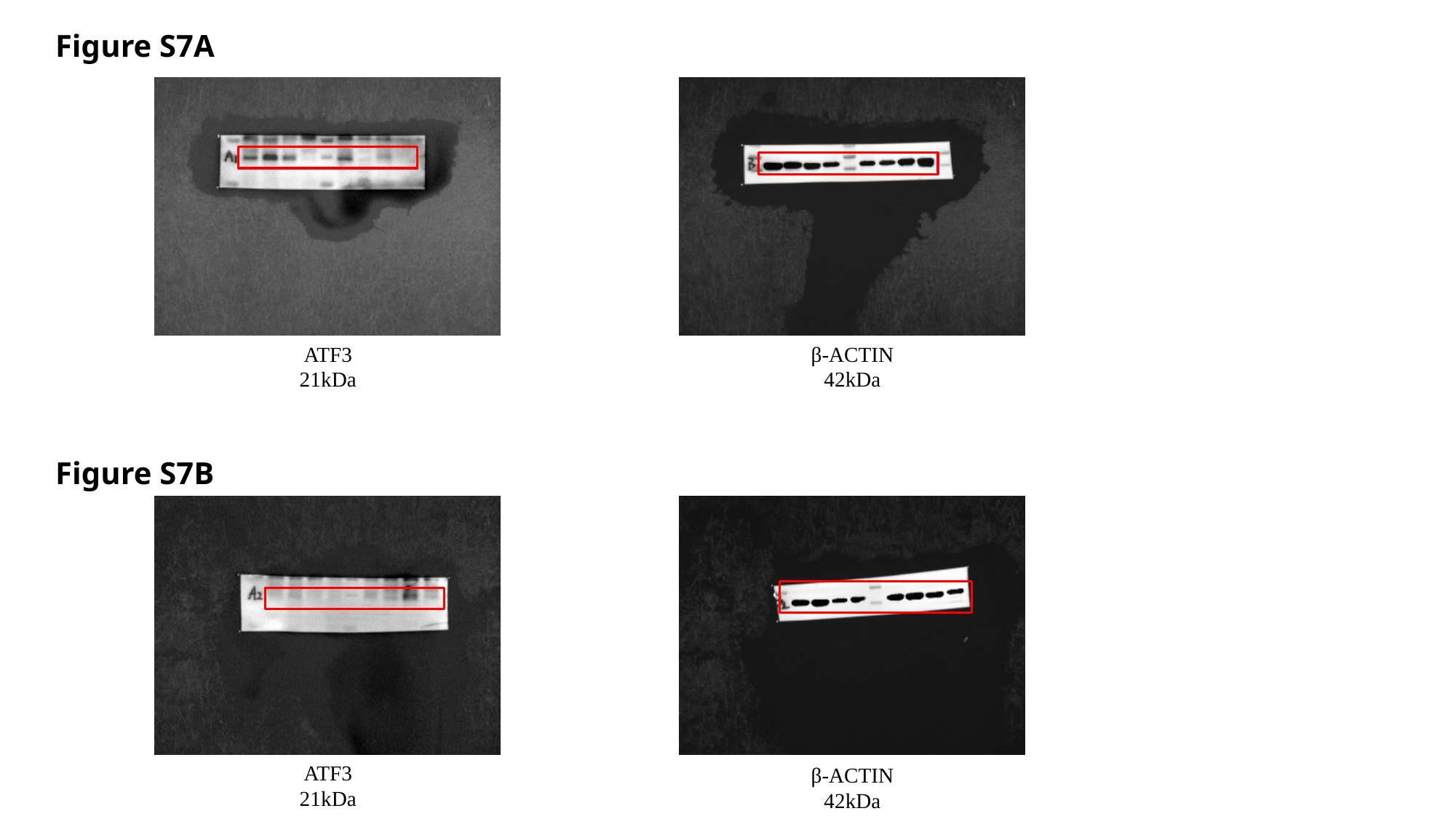

Figure S7A
ATF3
21kDa
β-ACTIN
42kDa
Figure S7B
ATF3
21kDa
β-ACTIN
42kDa
