# Supplementary material for: METTL1 drives tumor progression of bladder cancer via degrading ATF3 mRNA in an m7G-modified miR-760-dependent manner
Source: Cell Death Discov. 2022 Nov 17;8:458. doi: 10.1038/s41420-022-01236-6 (PMC9672058; doi:10.1038/s41420-022-01236-6)
Supplement: Supplementary file 3 — Supplementary Figure Legends [file 41420_2022_1236_MOESM3_ESM.docx]

**Supplementary Figure**

Figure S1. Upregulated METTL1 promotes BCa progression. Related to Figure 1. A. Analysis BCa samples in TCGA database showing expression of METTL1 in bladder cancer with metastasis group was higher than bladder cancer without metastasis group (p-value: 2.022e-3). B. Representative images of IHC staining of METTL1 in normal urothelial tissue and BCa tissue from Human Protein Atlas. Red dotted line was boundary of normal urothelial layer. Red arrows pointed to normal urothelial layer. Yellow arrows pointed to tumor tissues.

Figure S2. Wound healing assay in UM-UC3. Related to Figure 2 and Figure 6. A. Wound healing assay showed impaired migration ability of METTL1 depleted UM-UC3 cells. A representative experiment of three independent biological replicates is shown. B. Wound healing assay suggested enhanced migration ability by METTL1 overexpression in UM-UC3 cells. A representative experiment of three independent biological replicates is shown. C. Wound healing assay indicating the enhanced migration ability of cells overexpressed miR-760 in UM-UC3 cells. A representative experiment of three independent biological replicates is shown.

Figure S3. The suppressive role of three shMETTL1 sequences in proliferation and migration of BCa. Related to Figure 3. A. METTL1 was depleted by three shMETTL1s and shMETTL1-2 was the most effective. GAPDH was used for the normalization control. A representative experiment of three independent biological replicates is shown. B. shMETTL1-2 was the most effective sequence to inhibit proliferation of T24 and UM-UC3. The average of six biological replicates ± SDs is shown (***p<0.001, two-tailed t test). C. shMETTL1-2 was the most effective sequence to impair the colony formation of T24 and UM-UC3. The average of three independent biological replicates ± SDs is shown (*p<0.05, **p<0.01), ***p<0.001, two-tailed t test). D. shMETTL1-2 was the most effective sequence to suppress the migration of T24 and UM-UC3. The average of three independent biological replicates ± SDs is shown (*p<0.05, **p<0.01, ***p<0.001, two-tailed t test).

Figure S4. METTL1 promotes pri-miR-760 processing in an m^7^G dependent manner. Related to Figure 4. A. RT-qPCR assay showing METTL1 depletion didn’t alter the expression of pri-miR-760. GAPDH was used for the normalization control. The average of three independent biological replicates ± SDs is shown. B. RT-qPCR assay showing depleted METTL1 decreased expression of pre-miR-760. U6 was used for the normalization control. The average of three independent biological replicates ± SDs is shown (**p<0.01, ***p<0.001, two-tailed t test). C. RIP-RT-qPCR assay showing pre-miR-760 was enriched by METTL1 antibody indicating METTL1 bond to pre-miR-760 in T24 and UM-UC3 cells. The average of three independent immunoprecipitation reactions ± SDs is shown (***p<0.001, two-tailed t test). D. DROSHA IP-RT-qPCR assay showed the decreased pri-miR-760 enriched by DROSHA specific antibody upon the METTL1 depletion, suggesting METTL1 regulated pri-miR-760 processing in UM-UC3 cells. The average of three independent immunoprecipitation reactions ± SDs is shown (**p<0.01, two-tailed t test).

Figure S5. Screening potential targets of METTL1/m^7^G/miR-760. Related to Figure 5. A. Expression levels of TMEM184B, ATXN1L, KCNB1 and SYNPO2 in primary tumors vs normal subjects and among stages from TCGA database. Statistical significance was determined by Student's t test: **p<0.01, ***p<0.001. B. RT-qPCR assay showed the relative mRNA expression of KCNB1, SYNPO2 and ATXN1L in METTL1 depleted cells. GAPDH was used for the normalization control. The average of three independent biological replicates ± SDs is shown (*p<0.05, **p<0.01, ***p<0.001, two-tailed t test). Results in red squares were consistent with mRNA-seq. C. RT-qPCR assay showed the relative mRNA expression of KCNB1, SYNPO2 and ATXN1L in miR-760 overexpressed cells. GAPDH was used for the normalization control. The average of three independent biological replicates ± SDs is shown (***p<0.001, two-tailed t test). Results in red squares were consistent with expectations.

Figure S6. Inhibited proliferation and migration ability induced by siMETTL1 was rescued by miR-760. Related to Figure 5. A. RT-qPCR assay showed overexpressed miR-760 in rescue assay. B. Decreased colony induced by siMETTL1 was rescued by miR-760. The average of three independent biological replicates ± SDs is shown (**p<0.01, two-tailed t test). C. Impaired migration ability induced by siMETTL1 was rescued by miR-760. The average of three independent biological replicates ± SDs is shown (***p<0.001, two-tailed t test).

Figure S7. Protein expression level of ATF3 in tissue samples with elevated ATF3 mRNA expression. Related to Figure 6. A. Representative western blot assay showed Protein expression level of ATF3 in tissue samples with elevated ATF3 mRNA expression. β-actin was used for the normalization control.

Table S1. Baseline information of 17 patients with BCa.

Table S2. The primers used in this study.

Table S3. Differential miRNAs in METTL1 depleted UM-UC3 cells.

Table S4. Differential mRNAs in METTL1 depleted UM-UC3 cells.
