# Supplementary figures and images for: METTL1 drives tumor progression of bladder cancer via degrading ATF3 mRNA in an m7G-modified miR-760-dependent manner
Source: Cell Death Discov. 2022 Nov 17;8:458. doi: 10.1038/s41420-022-01236-6 (PMC9672058; doi:10.1038/s41420-022-01236-6)

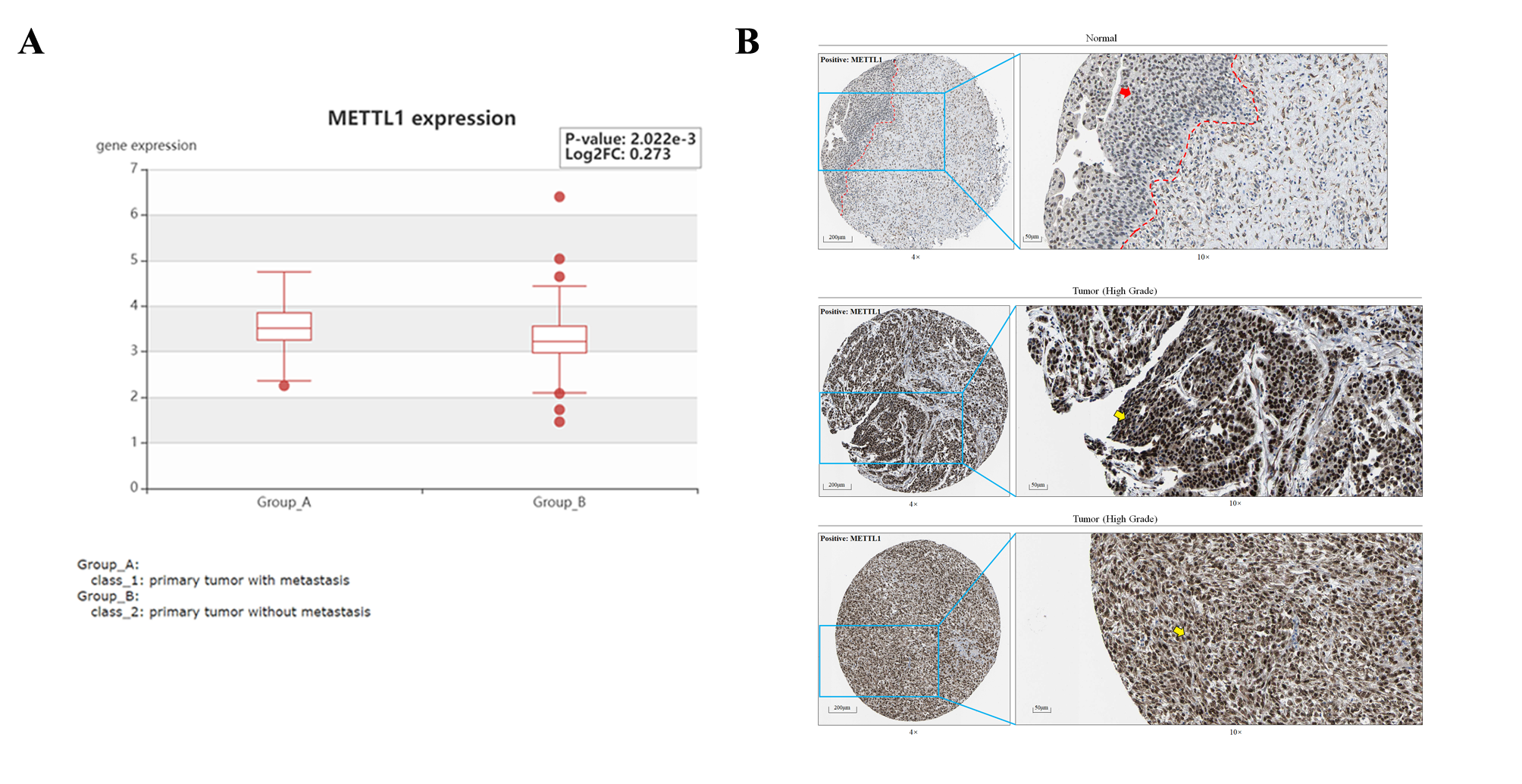

Supplement: Supplementary file 4 — Figure Supplementary 1 [file 41420_2022_1236_MOESM4_ESM.tif]

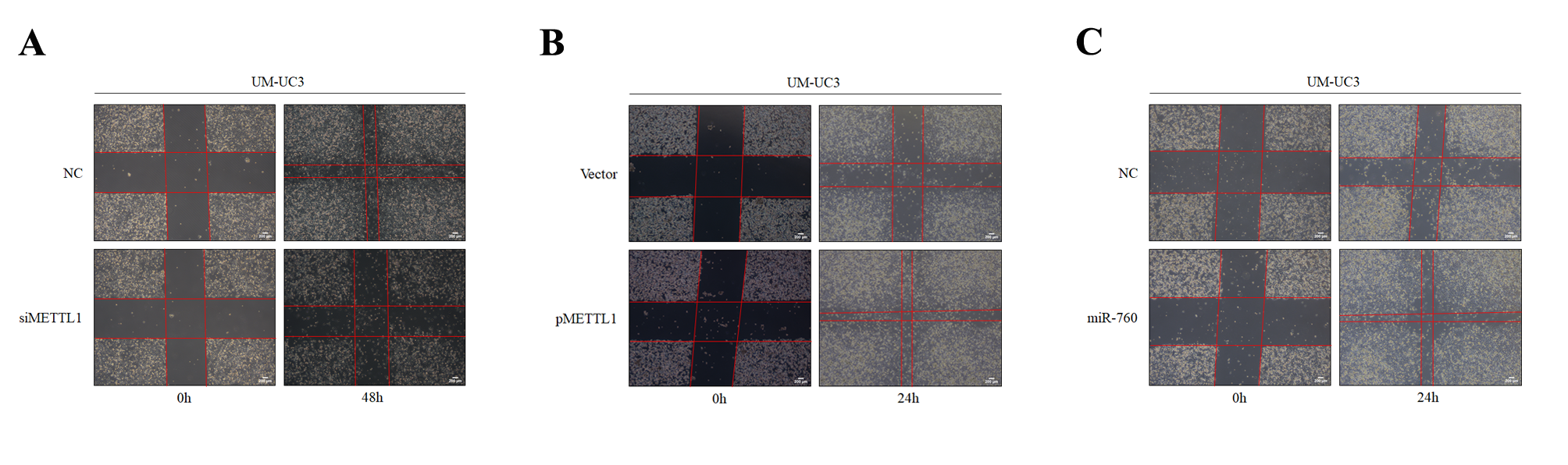

Supplement: Supplementary file 5 — Figure Supplementary 2 [file 41420_2022_1236_MOESM5_ESM.tif]

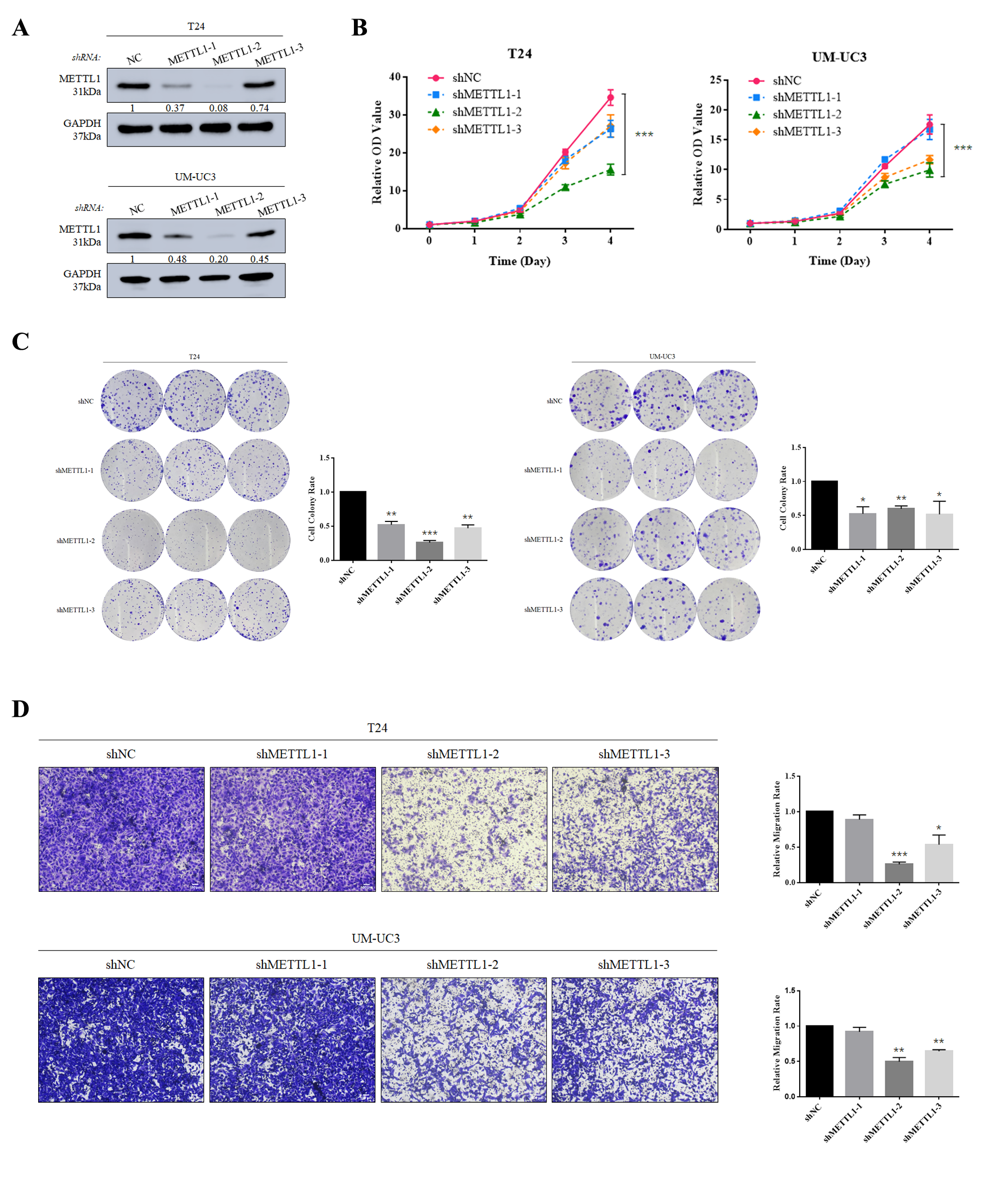

Supplement: Supplementary file 6 — Figure Supplementary 3 [file 41420_2022_1236_MOESM6_ESM.tif]

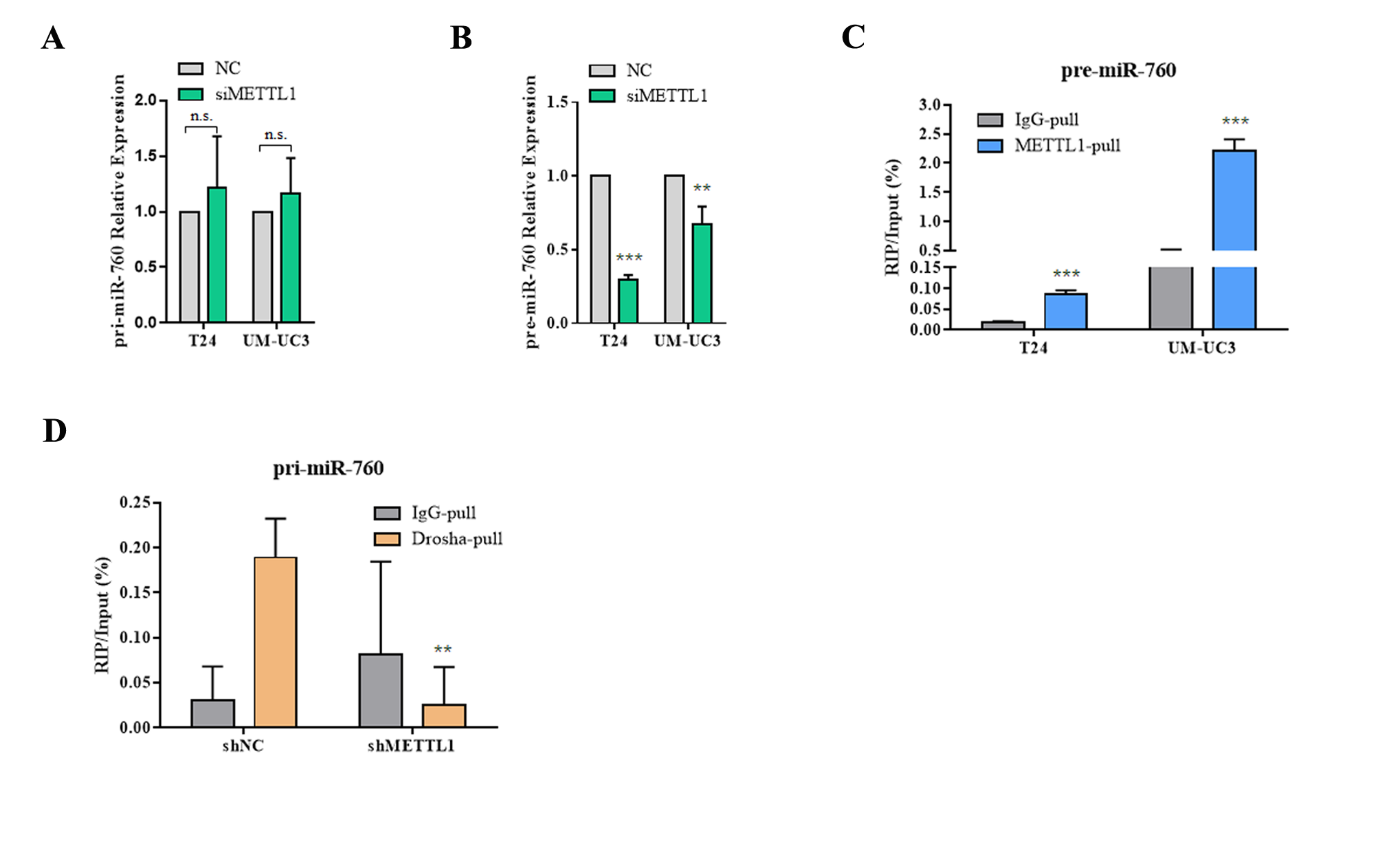

Supplement: Supplementary file 7 — Figure Supplementary 4 [file 41420_2022_1236_MOESM7_ESM.tif]

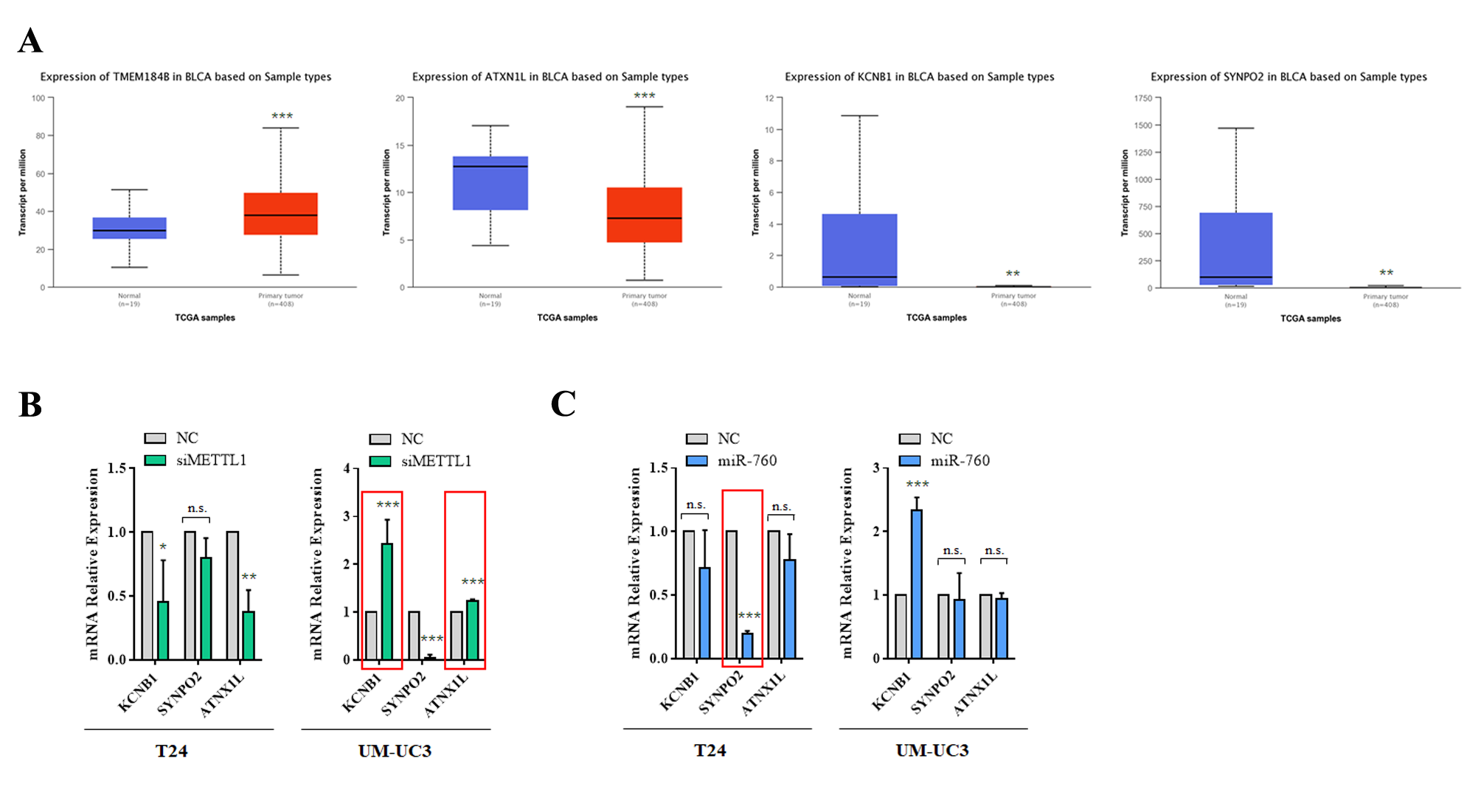

Supplement: Supplementary file 8 — Figure Supplementary 5 [file 41420_2022_1236_MOESM8_ESM.tif]

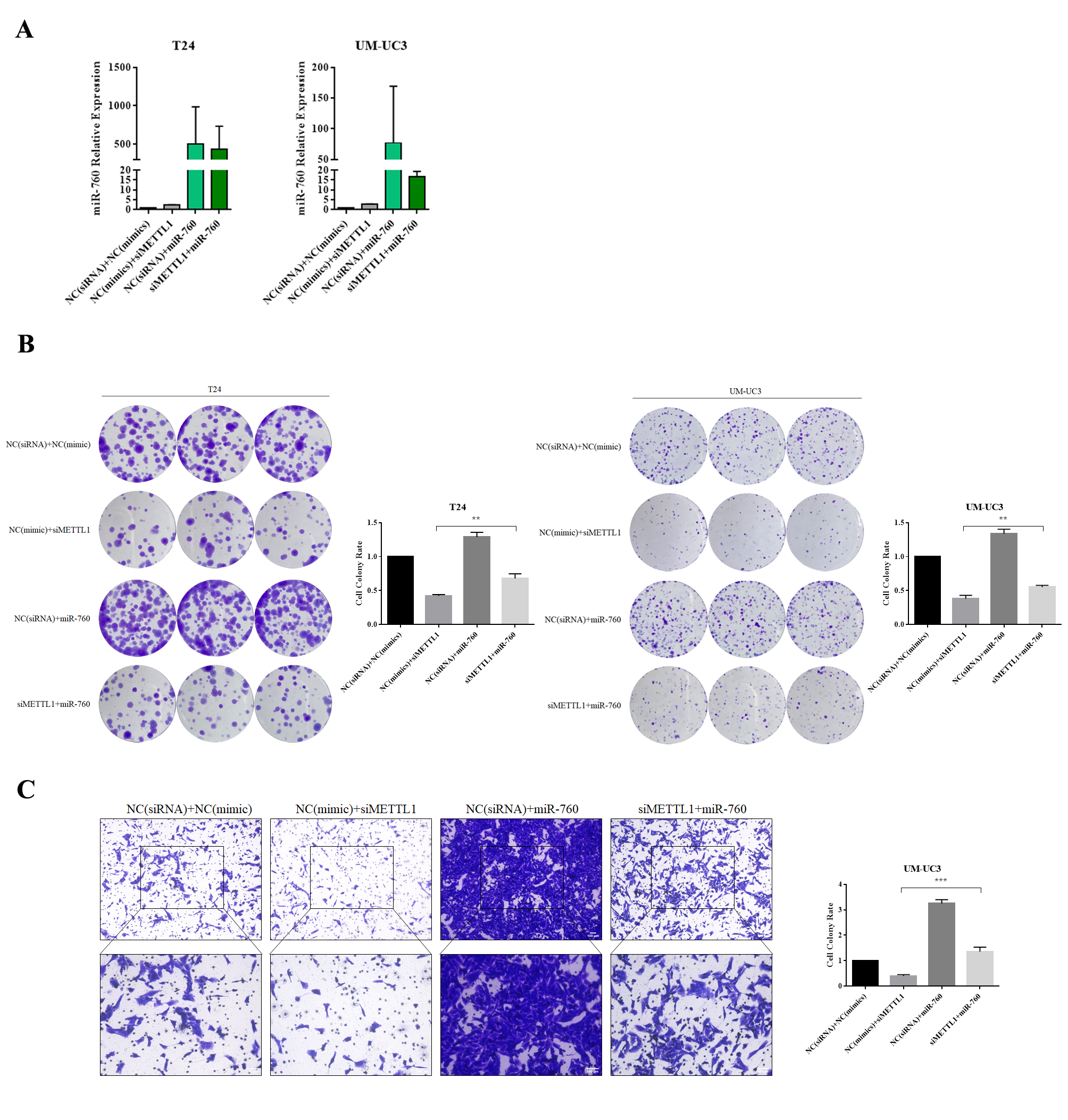

Supplement: Supplementary file 9 — Figure Supplementary 6 [file 41420_2022_1236_MOESM9_ESM.tif]

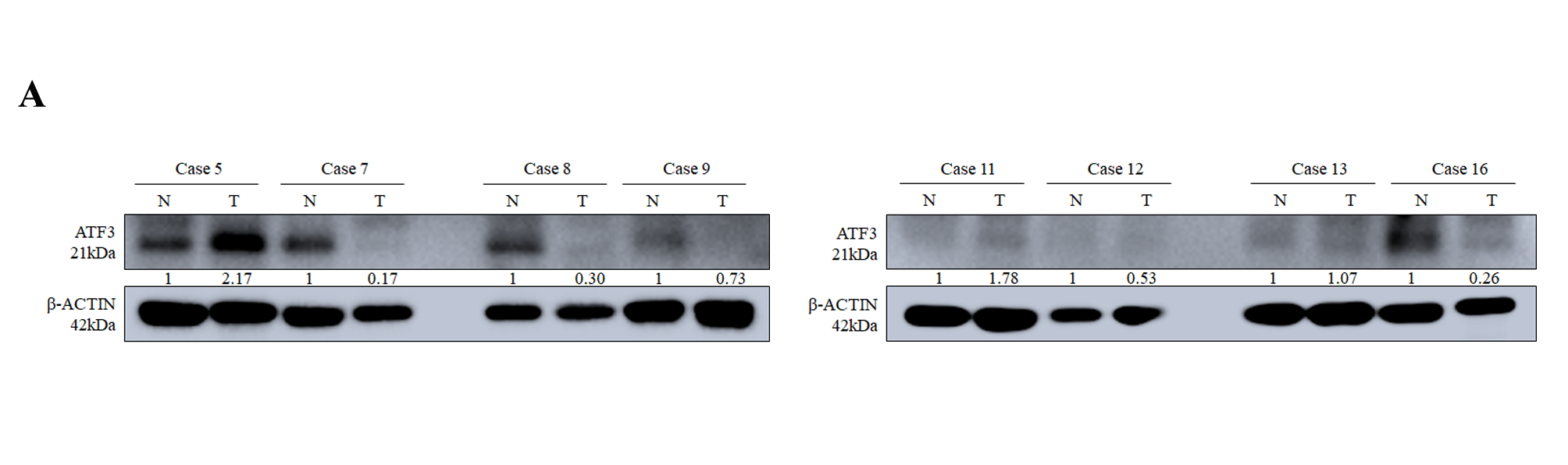

Supplement: Supplementary file 10 — Figure Supplementary 7 [file 41420_2022_1236_MOESM10_ESM.tif]
