# Supplementary material for: METTL1 drives tumor progression of bladder cancer via degrading ATF3 mRNA in an m7G-modified miR-760-dependent manner
Source: Cell Death Discov. 2022 Nov 17;8:458. doi: 10.1038/s41420-022-01236-6 (PMC9672058; doi:10.1038/s41420-022-01236-6)
Supplement: Supplementary file 11 — Table S1 [file 41420_2022_1236_MOESM11_ESM.docx]

| **Characteristic** | **Case number (percentage)** |
| --- | --- |
| Gender |  |
| Male | 15 (88.2%) |
| Female | 2 (11.8%) |
| Age |  |
| ＜60 | 3 (17.6%) |
| ＞60 | 14 (82.4%) |
| Stage |  |
| NMIBC (Ta/T1/Tis) | 10 (58.8%) |
| MIBC (T2-T4) | 7 (41.2%) |

Table S1. Baseline information of 17 patients with BCa
